# Deficient IFN Signaling by Myeloid Cells Leads to MAVS-Dependent Virus-Induced Sepsis

**DOI:** 10.1371/journal.ppat.1004086

**Published:** 2014-04-17

**Authors:** Amelia K. Pinto, Hilario J. Ramos, Xiaobo Wu, Shilpa Aggarwal, Bimmi Shrestha, Matthew Gorman, Kristin Y. Kim, Mehul S. Suthar, John P. Atkinson, Michael Gale Jr, Michael S. Diamond

**Affiliations:** 1 Department of Medicine, Washington University School of Medicine, St. Louis, Missouri, United States of America; 2 Department of Immunology, University of Washington School of Medicine, Seattle, Washington, United States of America; 3 Department of Pathology & Immunology, Washington University School of Medicine, St. Louis, Missouri, United States of America; 4 Department of Pediatrics, Emory Vaccine Center, Children's Healthcare of Atlanta, Emory University, Atlanta, Georgia, United States of America; 5 Molecular Microbiology, Washington University School of Medicine, St. Louis, Missouri, United States of America; Harvard Medical School, United States of America

## Abstract

The type I interferon (IFN) signaling response limits infection of many RNA and DNA viruses. To define key cell types that require type I IFN signaling to orchestrate immunity against West Nile virus (WNV), we infected mice with conditional deletions of the type I IFN receptor (IFNAR) gene. Deletion of the *Ifnar* gene in subsets of myeloid cells resulted in uncontrolled WNV replication, vasoactive cytokine production, sepsis, organ damage, and death that were remarkably similar to infection of *Ifnar* ^−/−^ mice completely lacking type I IFN signaling. In *Mavs^−/−^*×*Ifnar^−/−^* myeloid cells and mice lacking both *Ifnar* and the RIG-I-like receptor adaptor gene *Mavs*, cytokine production was muted despite high levels of WNV infection. Thus, in myeloid cells, viral infection triggers signaling through MAVS to induce proinflammatory cytokines that can result in sepsis and organ damage. Viral pathogenesis was caused in part by massive complement activation, as liver damage was minimized in animals lacking complement components C3 or factor B or treated with neutralizing anti-C5 antibodies. Disease in *Ifnar* ^−/−^ and CD11c Cre^+^ *Ifnar* ^f/f^ mice also was facilitated by the proinflammatory cytokine TNF-α, as blocking antibodies diminished complement activation and prolonged survival without altering viral burden. Collectively, our findings establish the dominant role of type I IFN signaling in myeloid cells in restricting virus infection and controlling pathological inflammation and tissue injury.

## Author Summary

Although it is well established that the interferon (IFN) signaling pathway restricts infection by many viruses, the key cell types *in vivo* that contribute to this process remain poorly characterized. To address this question in the context of West Nile virus (WNV) pathogenesis, we infected mice that specifically delete the type I IFN receptor gene (*Ifnar*) in subsets of myeloid cells, including dendritic cells and macrophages. Remarkably, mice lacking *Ifnar* expression only in myeloid cell subsets rapidly developed a sepsis-like syndrome that was characterized by enhanced WNV infection and visceral organ injury and caused by massive proinflammatory cytokine production and complement activation. By using additional gene targeted deletion mice, we show that WNV infection triggered signaling through the RIG-I like receptor adaptor protein MAVS to cause complement activation, sepsis, and tissue damage. Indeed, liver damage was minimized in animals lacking specific complement components, or treated with neutralizing anti-complement or anti-TNF-α antibodies. Our results establish how type I IFN signaling in dendritic cells and macrophages restricts infection, controls inflammatory cascades, and prevents pathogenesis *in vivo*.

## Introduction

Type I interferons (IFN) are a family of antiviral cytokines that are produced early in response to viral infection [1]. RNA intermediates of viral replication are recognized by cytosolic and endosomal pattern recognition receptors (PRR), such as RIG-I-like receptors (RLR) or Toll-like receptors (TLR), which signal through adaptor molecules (e.g., MAVS, TRIF, and MyD88) and transcription factors (e.g., IRF-3 and IRF-7) to induce type I IFN expression and secretion. Type I IFNs bind to a heterodimeric receptor (IFNAR) and mediate pleiotropic effects downstream of a canonical Janus kinase (JAK)-Signal transducers and activators of transcription (STAT) signaling pathway. This results in the induction of antiviral IFN-stimulated genes (ISGs), activation of antigen-presenting cells, and regulation of cytokine and chemokine production (reviewed in [2]). Although IFNAR is expressed on all nucleated cells, individual cell types may respond differently to signaling by type I IFN, as evidenced by overlapping yet distinct transcriptional programs [3], [4].

CD11c^+^ cells are integral members of the mammalian innate and adaptive immune response. In the mouse, CD11c is expressed highly on dendritic cell (DC) subsets (CD4^+^, CD8α^+^, and CD103^+^), some macrophage (MØ) populations, and on selected CD8^+^ T cell subsets [5]–[7]. CD11c^+^ cells are professional antigen presenting cells that respond to viral infection through a number of PRR, including the RLRs. CD11c^+^ DCs process and present antigens, express co-stimulatory molecules, and secrete cytokines and chemokines that regulate cell migration, leukocyte recruitment, and activation of adaptive immunity [7].

West Nile virus (WNV) is a member of the *Flaviviridae* family of positive-stranded enveloped RNA viruses and causes neuroinvasive disease in humans and other vertebrate animals [8]. Mosquitoes transmit WNV and in the skin CD11c^+^ cells and keratinocytes are believed to be initial targets of infection [9], [10]. Infected DCs migrate to the draining lymph node (LN) where viral replication ensues, resulting in viremia and spread to other peripheral organs [11]. Viral replication in the skin and LN induces a local and systemic type I IFN response, which is critical for limiting WNV replication and preventing dissemination to the brain and spinal cord. Indeed, subcutaneous WNV infection of *Ifnar* ^−/−^ mice results in a rapidly fatal infection, which is associated with high viremia and altered tissue and cellular tropism compared to wild-type (WT) mice [12], [13]. Analogously, *Mavs^−/−^* mice, which lack RLR signaling, exhibit increased susceptibility to WNV infection in many tissues [14]. Recent reports also have observed that the control and regulation of WNV infection requires IL-1ß production [15]–[17], and that inflammasome activation synergizes with type I IFN signaling to suppress WNV replication.

The significance of cell type-specific responses to IFN *in vivo* in the context of the control of viral pathogenesis is not well understood. Prior experiments with WNV infection suggested that type I IFN signaling has distinct temporal functions in the development of adaptive immunity [18]. The generation of cell type-specific conditional deletions of *Ifnar* has allowed its role on specific cell types to be analyzed in the context of infectious, inflammatory, or neoplastic disease [19]. Studies have shown an enhanced susceptibility to mouse hepatitis (MHV) and norovirus (MNoV) infection in animals with conditional *Ifnar* deletions, and this was associated with greater viral burden and decreased survival compared to WT animals [20]–[22].

Here, we evaluated WNV pathogenesis in mice lacking *Ifnar* expression in CD11c^+^ cells (CD11c Cre^+^ *Ifnar* ^f/f^ mice) or MØ/monocytes/granulocytes (LysM Cre^+^ *Ifnar* ^f/f^ mice). Remarkably, deletion of *Ifnar* in either CD11c^+^ or MØ/monocyte/granulocyte cells resulted in severe WNV disease that essentially copied the phenotype of the complete *Ifnar* ^−/−^ mice. Thus, the dominant antiviral effects *in vivo* of type I IFN signaling against WNV occur in myeloid cell types. Analysis of WNV-infected mice revealed preferential infection of *Ifnar* ^−/−^ myeloid cells, and this resulted in a syndrome of “cytokine storm”, which was associated with liver and kidney damage, and rapid death. Immunopathogenesis in WNV-infected *Ifnar* ^−/−^ and CD11c Cre^+^ *Ifnar* ^f/f^ mice animals was mitigated by exogenous administration of TNF-α blocking antibodies, and the sepsis syndrome was associated with massive alternative pathway complement activation as tissue damage was improved in mice lacking the complement components C3 or factor B. Our experiments suggest that high levels of viral replication in WNV-infected *Ifnar* ^−/−^ myeloid cells can trigger uncontrolled production of proinflammatory cytokines and pathological complement induction and activation, which together contribute to a sepsis-like syndrome.

## Results

### CD11c Cre^+^ *Ifnar* ^f/f^, LysM Cre^+^ *Ifnar* ^f/f^, and *Ifnar* ^−/−^ mice are vulnerable to WNV infection

*In vivo*, WNV preferentially infects myeloid cells in peripheral tissues and neurons in the brain and spinal cord [23]–[25]. Given that DCs are targets of WNV infection, produce antiviral cytokines, and shape adaptive immunity, we hypothesized that type I IFN signaling in these cells orchestrates protection against WNV. To assess the role of type I IFN receptor signaling on DCs, we utilized CD11c Cre^+^ *Ifnar* ^f/f^ mice, in which IFNAR expression is markedly decreased on CD4^+^ and CD8α^+^ DCs but maintained on other hematopoietic cells, including neutrophils, natural killer, T and B cells ([26] and data not shown).

**Figure 1:**
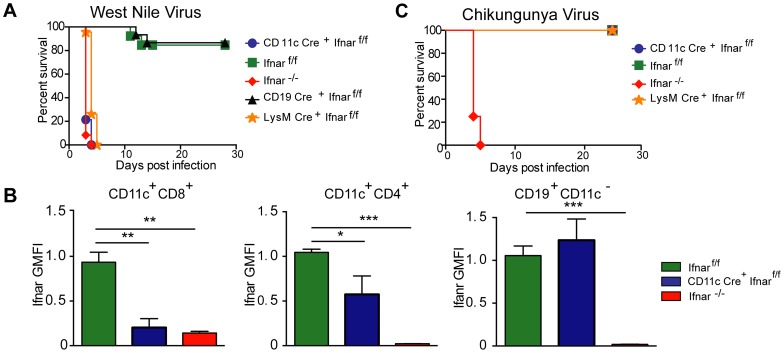
CD11c Cre^+^ *Ifnar* ^f/f^, LysM Cre^+^ *Ifnar* ^f/f^ and *Ifnar* ^−/−^ mice are vulnerable to WNV infection. **A**. Survival of eight- to ten-week-old mice after inoculation with 10^2^ PFU of WNV by footpad injection. Survival differences were statistically significant between *Ifnar* ^f/f^ and CD11c Cre^+^ *Ifnar* ^f/f^ (***, *P*<0.0001), *Ifnar* ^f/f^ and LysM Cre^+^ *Ifnar* ^f/f^ (***, *P*<0.0001), and *Ifnar* ^f/f^ and *Ifnar* ^−/−^ mice (***, *P*<0.0001) but not between Cre^−^ *Ifnar* ^f/f^ (*Ifnar* ^f/f^) and CD19 Cre^+^ *Ifnar* ^f/f^ (*n* = 20, CD11c Cre^+^ *Ifnar* ^f/f^, *n* = 18, LysM Cre^+^ *Ifnar* ^f/f^ *n* = 15, *Ifnar* ^f/f^, *n* = 15 *Ifnar* ^−/−^, and *n* = 8 CD19 Cre^+^ *Ifnar* ^f/f^). **B**. Analysis of IFNAR expression from hematopoietic cells of *Ifnar* ^−/−^, CD11c Cre^+^ *Ifnar* ^f/f^, and Cre^−^ *Ifnar* ^f/f^ infected mice (*n* = 6 to 8 per group). Blood was harvested 48 hours after WNV infection, and cells were stained with MAbs against IFNAR, CD11c, CD3, CD8, CD4, and CD19. The relative staining of IFNAR on CD11c^+^CD8^+^CD3^−^, CD11c^+^CD4^+^CD3^−^ and CD19^+^CD11c^−^ CD3^−^ is shown. The data is pooled from two independent experiments after normalization of Ifnar expression on Cre^−^ *Ifnar* ^f/f^ cells within a given experiment. **C**. Survival of eight week-old mice infected with 10 PFU of CHIKV by footpad injection. Survival differences were statistically significant between *Ifnar* ^f/f^ and *Ifnar* ^−/−^ mice (***, *P*<0.0001) but not between *Ifnar* ^f/f^ and CD11c Cre^+^ *Ifnar* ^f/f^ or *Ifnar* ^f/f^ and LysM Cre^+^ *Ifnar* ^f/f^ (*n* = 8, CD11c Cre^+^ *Ifnar* ^f/f^, *n* = 8 LysM Cre^+^ *Ifnar* ^f/f^, *n* = 12 *Ifnar* ^f/f^, and *n* = 12 *Ifnar* ^−/−^ mice). The CD11c-Cre recombinase deletes *Ifnar* on greater than 95% of conventional CD11c^high^ dendritic cells and ∼50% of plasmacytoid dendritic cells [88]. The LysM-Cre recombinase deletes *Ifnar* on mature MØ, granulocytes, and monocytes, with partial gene deletion (∼16%) in CD11c^+^ splenic dendritic cells [89].

Infection of *Ifnar* ^−/−^ mice with WNV resulted in 100% mortality with a mean survival time of 3 days, as reported previously [12]. Unexpectedly, infection of CD11c Cre^+^ *Ifnar* ^f/f^ mice resulted in essentially the same phenotype (**Fig. 1A**) with no difference in the mean survival time compared to *Ifnar* ^−/−^ mice. Although WNV-infected *Ifnar* ^−/−^ and CD11c Cre^+^ *Ifnar* ^f/f^ mice rapidly deteriorated, clinical signs of neuroinvasive disease including limb paralysis, seizures, ataxia, or sustained tremors were not apparent. *Ifnar* ^−/−^ and CD11c Cre^+^ *Ifnar* ^f/f^ mice were more vulnerable to WNV infection than Cre^−^ *Ifnar* ^f/f^ littermate controls or CD19 Cre^+^ *Ifnar* ^f/f^ mice, which lack IFNAR expression only on B cells. The decreased expression of IFNAR on CD11c Cre^+^ *Ifnar* ^f/f^ cells was confirmed in mice following WNV infection (**Fig. 1B**). Thus, the loss of IFNAR expression on CD11c^+^ cells resulted in a clinical phenotype after WNV infection that was nearly identical to a deletion of IFNAR on all cells. When we repeated a subset of experiments with LysM Cre^+^ *Ifnar* ^f/f^ mice, which delete IFNAR expression on MØ, monocytes, and granulocytes (data not shown), a similar rapid death phenotype was observed after WNV infection (**Fig. 1A**), with a minimally longer survival time (mean of 3.5 days) compared to the *Ifnar* ^−/−^ or CD11c Cre^+^ *Ifnar* ^f/f^ mice.

To determine whether the susceptibility phenotype of the CD11c Cre^+^ *Ifnar* ^f/f^ mice could be generalized to other arthropod-borne viruses, we infected animals with chikungunya virus (CHIKV), an unrelated arthritogenic alphavirus (**Fig. 1C**). CHIKV preferentially targets myoblasts, fibroblasts, and some MØ populations but is not reported to infect DCs [27]. As seen previously [28], *Ifnar* ^−/−^ mice infected with CHIKV succumbed to infection within 4 to 5 days. In contrast, the CD11c Cre^+^ *Ifnar* ^f/f^, LysM Cre^+^ *Ifnar* ^f/f^, or the Cre^−^ *Ifnar* ^f/f^ littermate control mice failed to develop lethal CHIKV infection. Thus, selective deletion of IFNAR expression on CD11c^+^ or other myeloid cells did not make mice vulnerable to other viruses, even if a complete gene deletion did; these results are consistent with studies with the coronavirus MHV, which showed a partial lethality phenotype in CD11c Cre^+^ *Ifnar* ^f/f^ mice [20].

**Figure 2:**
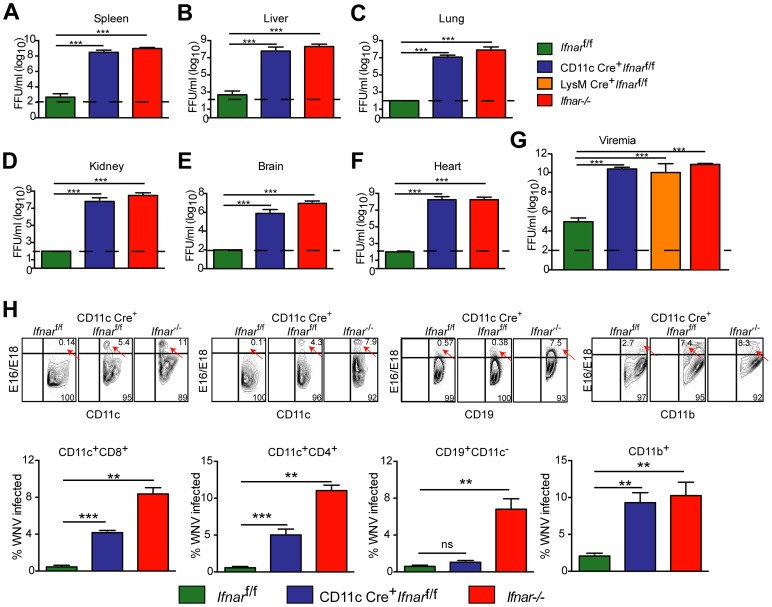
Viral infection in WNV-infected CD11c Cre^+^ *Ifnar* ^f/f^, Cre^−^ *Ifnar* ^f/f^, and *Ifnar* ^−/−^ mice. **A–G**. Viral burden in peripheral and CNS tissues after WNV infection. Eight- to ten-week-old mice were inoculated with 10^2^ PFU of WNV by footpad injection. Levels of infectious virus in the (**A**) spleen, (**B**) liver, (**C**) lung, (**D**) kidney, (**E**) brain, (**F**) heart, and (**G**) serum were determined from samples harvested 48 hours post-infection using focus-forming assays. Data are shown as FFU per mg of tissue or per ml of serum for six to eight mice per time point. The dotted line represents the limit of sensitivity of the assay and error bars indicate standard deviation (SD). Asterisks indicate values that are statistically significant (***, *P*<0.0001) compared to Cre^−^ *Ifnar* ^f/f^ mice. **H**. Blood was harvested 48 hours after WNV infection, and cells were stained with MAbs against CD11c, CD11b, CD3, CD8, CD4, GR-1, and CD19 followed by intracellular staining against the WNV E protein with a combination of two anti-WNV MAbs (WNV E16 and WNV E18). A representative contour plot is provided and shows intracellular WNV antigen levels (red arrows) in cells at 48 hours after inoculation. The percentage of WNV-infected cells for each cell population from each group is shown in the graphs immediately below.

We next assessed whether the loss of IFNAR expression on CD11c^+^ cells impacted tissue viral burden to the same extent as that of *Ifnar* ^−/−^ mice. High titers (10^6^ to 10^9^ FFU/mg) of WNV were detected in the spleen, liver, lung, kidney, brain, and heart of *Ifnar* ^−/−^ and CD11c Cre^+^ *Ifnar* ^f/f^ mice at 48 hours post infection (**Fig. 2A–F**). As expected, greater infection was observed in *Ifnar* ^−/−^ and CD11c Cre^+^ *Ifnar* ^f/f^ mice compared to Cre^−^ *Ifnar* ^f/f^ littermate controls. Similar trends in WNV infection were observed in the serum of *Ifnar* ^−/−^, CD11c Cre^+^ *Ifnar* ^f/f^, and LysM Cre^+^ *Ifnar* ^f/f^ mice at 48 hours (**Fig. 2G**). Flow cytometric analysis of immune cells from the blood of infected *Ifnar* ^−/−^ mice revealed intracellular WNV antigen in CD11c^+^ CD8^+^ cells, CD11c^+^ CD4^+^ cells, CD11b^+^ cells, and some CD19^+^ cells (**Fig. 2H**), consistent with an earlier report [12]. In infected CD11c Cre^+^ *Ifnar* ^f/f^ mice, WNV antigen was detectable in ∼4 to 6 percent of CD11c^+^ cells, and in ∼8 to 13 percent of CD11b^+^ cells that co-expressed varying amounts of CD11c. In WNV-infected Cre^−^ *Ifnar* ^f/f^ mice, viral antigen in CD11c^+^ cells from the blood was near the threshold of detection at 48 hours after infection. Thus, a loss of IFNAR signaling in subsets of CD11c^+^ cells created a more permissive intracellular environment for WNV replication.

### Pathological analysis of tissues from WNV-infected CD11c Cre^+^ *Ifnar* ^f/f^ mice

**Figure 3:**
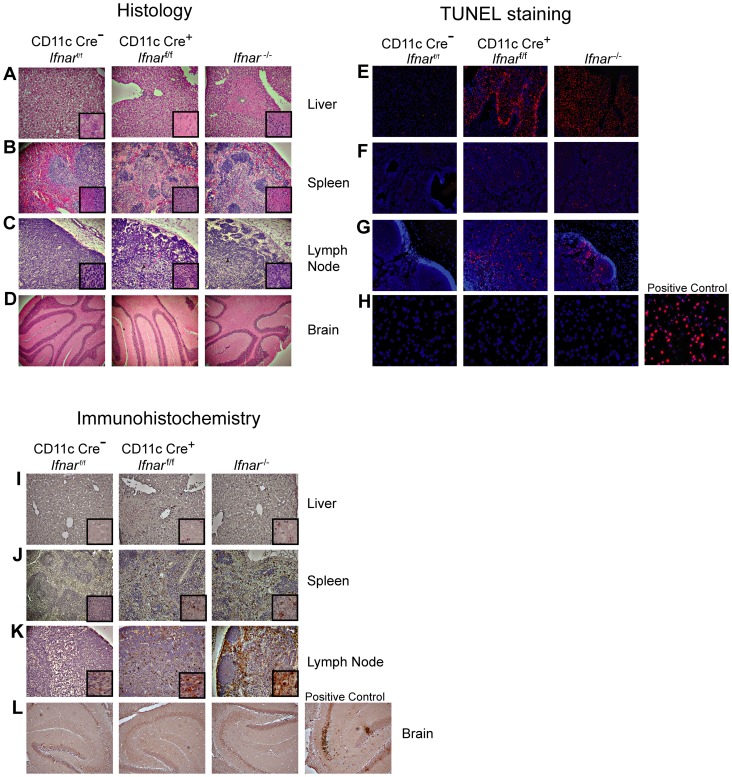
Tissue analysis of WNV-infected CD11c Cre^+^ *Ifnar* ^f/f^ mice. **A–C**. Histological analysis of the (**A**) liver, (**B**) spleen, (**C**) LN, and (**D**) brain of Cre^−^ *Ifnar* ^f/f^ (*Ifnar* ^f/f^), CD11c Cre^+^ *Ifnar* ^f/f^, and *Ifnar* ^−/−^ mice. Representative images are shown 48 hours after WNV infection of organs from five to six mice for each group. Inset images show staining at a higher magnification (100×). **E–H**. TUNEL staining of (**E**) liver, (**F**) spleen (**G**) LN, and (**H**) brain of Cre^−^ *Ifnar* ^f/f^ (*Ifnar* ^f/f^), CD11c Cre^+^ *Ifnar* ^f/f^, and *Ifnar* ^−/−^ mice 48 hours after WNV inoculation. Cells were counter stained with DAPI. Representative images are shown from five or six mice for each group. **I–L**. Detection by IHC of WNV antigen in (**I**) liver, (**J**) spleen (**K**) LN, and (**L**) brain of Cre^−^ *Ifnar* ^f/f^ (*Ifnar* ^f/f^), CD11c Cre^+^ *Ifnar* ^f/f^, and *Ifnar* ^−/−^ mice 48 hours after infection. Representative images are shown of sections from three or four mice for each group. All sections were counterstained with hematoxylin; magnifications are 40×. Insets at higher magnification also are shown.

Necropsy at 48 hours post infection revealed gross macroscopic tissue damage including hemorrhages, infarcts, and edema in the liver, spleen, and LN of *Ifnar* ^−/−^ and CD11c Cre^+^ *Ifnar* ^f/f^ mice that was not evident in WNV-infected Cre^−^ *Ifnar* ^f/f^ mice (data not shown). Histological analysis of visceral organs from *Ifnar* ^−/−^ and CD11c Cre^+^ *Ifnar* ^f/f^ mice showed increased cellular infiltrates, coagulation necrosis, and tissue destruction after WNV infection of the liver, spleen, and draining LN when compared to the Cre^−^ *Ifnar* ^f/f^ mice. However, little to no cellular infiltrate or tissue destruction was observed in the brains of any of the mice at this early time point (**Fig. 3A–D**). Terminal deoxynucleotidyl transferase dUTP nick end labeling (TUNEL) analysis confirmed greater numbers of dead cells in the liver, spleen, and LN but not in the brains of *Ifnar* ^−/−^ and CD11c Cre^+^ *Ifnar* ^f/f^ mice (**Fig. 3E–H**). Immunohistochemistry (IHC) was performed to determine whether these phenotypes correlated with cellular infection. Despite the high titer (10^8^ FFU/mg) of infectious virus in liver homogenates (**Fig. 2B**), we observed relatively low levels of WNV antigen staining in hepatic sections from *Ifnar* ^−/−^ and CD11c Cre^+^ *Ifnar* ^f/f^ mice (**Fig. 3I**). We also failed to observe any obvious brain pathology in mice at 48 hours and this correlated with a paucity of viral antigen in tissue sections. In comparison, WNV antigen staining was more apparent in the draining LN and the spleen of *Ifnar* ^−/−^ and CD11c Cre^+^ *Ifnar* ^f/f^ mice compared to Cre^−^ *Ifnar* ^f/f^ mice (**Fig. 3J and K**). Combined with the flow cytometry data on cell subsets (**Fig. 2H**), our analysis suggests that the enhanced viral burden in *Ifnar* ^−/−^ and CD11c Cre^+^ *Ifnar* ^f/f^ mice was attributable to preferential infection of *Ifnar* ^−/−^ CD11c^+^ cells. The high viral titers in some organs (e.g., liver and brain) showing little WNV antigen staining by IHC might be due to infected CD11c^+^ *Ifnar* ^−/−^ cells that were retained in blood vessels; these animals showed a deteriorating clinical phenotype and were difficult to perfuse despite large volumes of saline administration. Overall, the histology revealed a high degree of tissue injury in lymphoid compartments of WNV-infected *Ifnar* ^−/−^ and CD11c Cre^+^ *Ifnar* ^f/f^ mice that reflected enhanced infection of cells lacking IFNAR expression. Tissue destruction in some organs, however, did not appear commensurate with the level of viral antigen present in the tissue parenchyma.

### Blood chemistry reveals extensive liver and kidney injury

**Figure 4:**
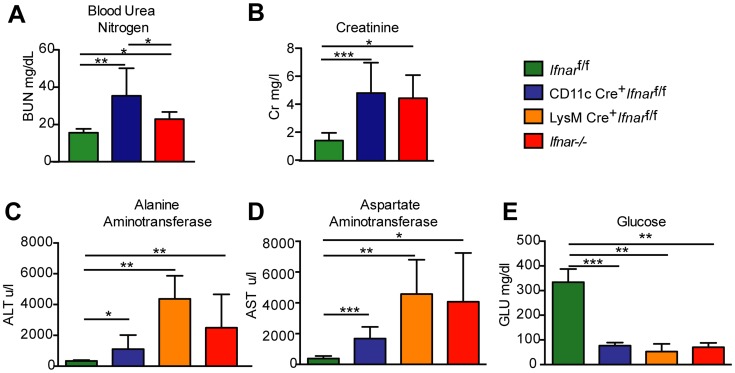
Blood chemistry reveals hepatic and renal injury in mice lacking Ifnar expression on myeloid cells. (**A**) Blood urea nitrogen (BUN), (**B**) creatinine (Cr), (**C**) Alanine aminotransferase (ALT), (**D**) aspartate aminotransferase (AST), and (**E**) glucose (GLU) levels were analyzed in the serum of *Ifnar* ^−/−^, CD11c Cre^+^ *Ifnar* ^f/f^, LysM Cre^+^ *Ifnar* ^f/f^, and Cre^−^ *Ifnar* ^f/f^ mice (*n* = 8 to 12 for each group) 48 hours after inoculation with 10^2^ PFU of WNV. Data are pooled from four independent experiments (*, *P*<0.05; **, *P*<0.01, ***, *P*<0.001).

To understand why *Ifnar* ^−/−^ and CD11c Cre^+^ *Ifnar* ^f/f^ mice succumbed so rapidly to WNV infection, we analyzed their blood chemistries. At 48 hours after WNV inoculation we observed increased levels of blood urea nitrogen (BUN) in *Ifnar* ^−/−^ and CD11c Cre^+^ *Ifnar* ^f/f^ mice (22.8 mg/dL and 32.4 mg/dL respectively, versus 15.5 mg/dL, *P*<0.02; **Fig. 4A**), serum creatinine (4.5 mg/L and 4.9 mg/L respectively, versus 1.5 mg/L, *P*<0.02; **Fig. 4B**), alanine aminotransferase (ALT) (2,495 u/L and 1,110 u/L respectively, versus 334 u/L, *P*<0.02; **Fig. 4C**), and aspartate aminotransferase (AST) (4,070 u/L and 1,676 u/L respectively, versus 382 u/L, *P*<0.02; **Fig. 4D**), and decreased levels of glucose (70 mg/dL and 77 mg/dL respectively, versus 334 mg/dL, *P*<0.004; **Fig. 4E**), compared to infected Cre^−^ *Ifnar* ^f/f^ mice. The elevated serum BUN and creatinine values indicate that WNV-infected *Ifnar* ^−/−^ and CD11c Cre^+^ *Ifnar* ^f/f^ mice experienced acute renal injury. The elevated liver enzymes (ALT and AST) reflect acute hepatic injury, which could be due to direct viral infection (although not seen by IHC), immune-mediated injury, or ischemia. The observed hypoglycemia may be secondary to depleted glycogen stores, impaired gluconeogenesis, or increased peripheral glucose utilization that occurs during infection or sepsis [29]. Similarly elevated serum levels of AST and ALT and decreased levels of glucose were observed in WNV-infected LysM Cre^+^ *Ifnar* ^f/f^ mice, compatible with a syndrome of acute hepatic injury (**Fig. 4C–E**).

### “Cytokine storm” in WNV-infected CD11c Cre^+^ *Ifnar* ^f/f^, LysM Cre^+^ *Ifnar* ^f/f^, and *Ifnar* ^−/−^ mice

**Figure 5:**
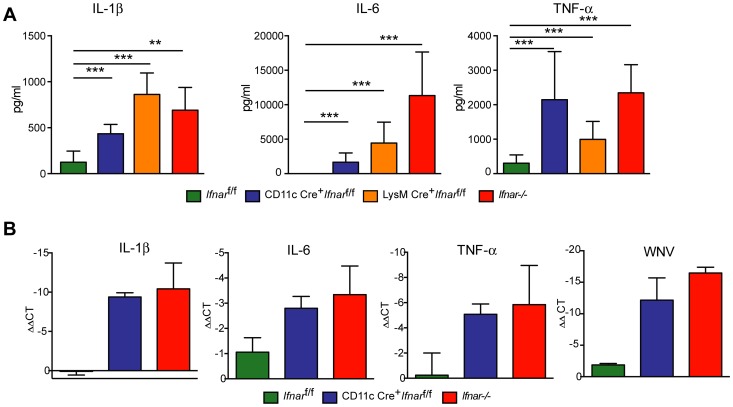
Serum cytokine levels in WNV-infected mice. **A**. *Ifnar* ^−/−^, CD11c Cre^+^ *Ifnar* ^f/f^, LysM Cre^+^ *Ifnar* ^f/f^, and Cre^−^ *Ifnar* ^f/f^ mice (*n* = 13 for each group) were infected 10^2^ PFU of WNV. 48 hours later, serum was collected and the concentration of IL-1ß, IL-6, and TNF-α was determined. Mean values ± SD are shown. **B**. Forty-eight hours after WNV infection splenic CD11c^+^ cells were isolated from *Ifnar* ^−/−^, CD11c Cre^+^ *Ifnar* ^f/f^, and Cre^−^ *Ifnar* ^f/f^ mice by positive selection with antibody-coated magnetic beads (*n* = 3 for each group). Total RNA was prepared and qRT-PCR was used to determine the amount of IL-1ß, IL-6, TNF-α, and WNV E RNA. The mean values ± SD are shown. The data is expressed as fold-increase in mRNA relative to samples from WNV-infected Cre^−^ *Ifnar* ^f/f^ mice.

To understand further the basis of organ damage in *Ifnar* ^−/−^, LysM Cre^+^ *Ifnar* ^f/f^, and CD11c Cre^+^ *Ifnar* ^f/f^ mice after WNV infection, we measured serum levels of 23 pro- and anti-inflammatory cytokines and chemokines using a Bioplex assay (**Fig. 5A** **and** **Table 1**). At 24 hours after infection, we detected no difference in cytokine levels among Cre^−^ *Ifnar* ^f/f^, *Ifnar* ^−/−^, and CD11c Cre^+^ *Ifnar* ^f/f^ mice. However, one day later, we observed increased levels of most proinflammatory cytokines in the *Ifnar* ^−/−^ and CD11c Cre^+^ *Ifnar* ^f/f^ mice when compared to the Cre^−^ *Ifnar* ^f/f^ mice; 22 of the 23 cytokines in the panel were elevated in mice lacking IFNAR expression on CD11c^+^ cells (**Table 1**). Examination of the cytokine profile in the serum of LysM Cre^+^ *Ifnar* ^f/f^ 48 hours after WNV infection also revealed an increase in 20 of the 23 measured cytokines and chemokines (**Fig. 5A** **and** **Table 1**). We detected 2 to 1,600 fold increases (*P*<0.0001) in levels of individual proinflammatory cytokines after WNV infection, which included vasoactive (e.g., TNF-α) and inflammasome-generated (e.g., IL-1ß) cytokines. The massive increase in multiple proinflammatory cytokines after WNV infection in *Ifnar* ^−/−^and CD11c or LysM Cre^+^ *Ifnar* ^f/f^ mice coupled with renal and hepatic injury and low viral antigen staining in tissues suggested a picture of sepsis due to “cytokine storm”. In comparison, relatively small increases in only six of the cytokines were observed in CHIKV infected *Ifnar* ^−/−^and Cre^−^ *Ifnar* ^f/f^ mice (**Fig. S1** and **Table S1**).

**Table 1:** Cytokine levels in serum of *Ifnar* ^−/−^, CD11c Cre^+^ *Ifnar* ^f/f^, LysM Cre^+^ *Ifnar* ^f/f^, and Cre^−^ *Ifnar* ^f/f^ mice during WNV infection.

	Cre-*Ifnar* ^f/f^		CD11c Cre^+^ *Ifnar* ^f/f^			LysM Cre^+^ *Ifnar* ^f/f^			*Ifnar^−/−^*		
Cytokine	pg/ml	SD	pg/ml	SD	P value	pg/ml	SD	P value	pg/ml	SD	P value
IL-1α	7.14	5.28	85.6	28.1	<0.0001	130	64.3	<0.0001	108	41.6	<0.0001
IL-1β	125	102	434	121	<0.0001	862	234	0.0001	690	248	0.0054
IL-2	7.03	5.45	26.2	20.9	0.0009	91.1	22.3	<0.0001	39.7	36.5	0.0003
IL-3	1.41	1.29	10.9	7.92	<0.0001	29.8	12.0	<0.0001	19.8	16.2	<0.0001
IL-4	27.8	19.2	113	59.3	0.0081	20.2	12.3	ns	154	54.6	0.0081
IL-5	6.04	5.20	109	49.8	<0.0001	117	55.6	<0.0001	103	100	<0.0001
IL-6	5.16	5.58	1655	1358	<0.0001	4434	3033	<0.0001	11325	6322	<0.0001
IL-9	384	160	957	226	0.0043	892	453	ns	1378	521	0.0043
IL-10	170	186	689	595	0.0012	303	127	ns	380	335	0.014
IL-12p40	607	415	4633	2873	<0.0001	2542	888	0.0001	6832	2814	<0.0001
IL-12p70	32.8	24.4	227	225	<0.0001	873	379	<0.0001	563	585	<0.0001
IL-13	119	142	313	241	0.0044	449	175	0.0008	464	325	0.0008
IL-17	34	26.5	51.0	28.4	ns	114	81.1	0.0012	60.1	39.7	ns
Eotaxin	126	144	1651	1155	0.0007	1401	797	0.0055	2277	998	0.0005
g-CSF	291	319	1.34e6	5.69e5	<0.0001	47988	25923	<0.0001	2.25e5	193040	<0.0001
GM-CSF	100	66.9	361	293	<0.0001	1156	352	<0.0001	604	607	<0.0001
IFN-γ	11.7	7.17	254	264	<0.0001	52.3	39.3	0.0055	240	162	<0.0001
KC	58.8	48.5	4919	2728	<0.0001	4923	4014	<0.0001	14352	11068	<0.0001
MCP-1	140	132	37705	14601	<0.0001	44084	20681	<0.0001	25176	16768	<0.0001
MIP-1α	8.49	5.87	177	62.3	<0.0001	272	147	<0.0001	181	82.5	<0.0001
MIP-1β	24.1	31.6	492	288	<0.0001	1404	568	<0.0001	575	500	<0.0001
RANTES	27	23.1	830	680	<0.0001	1152	556	<0.0001	1331	1400	<0.0001
TNF-α	306	241	2144	1397	<0.0001	995	519	0.0004	2345	816	<0.0001

*Ifnar* ^−/−^, CD11c or LysM Cre^+^ *Ifnar* ^f/f^, and Cre^−^ *Ifnar* ^f/f^ mice (*n* = 13 for each group) were infected with 10^2^ PFU of WNV. Forty-eight hours later serum was collected and cytokines were analyzed by bioplex assay. Mean (pg/ml) and standard deviations (SD) are shown and *P* values are compared to Cre^−^ *Ifnar* ^f/f^ mice. Data are pooled from four independent experiments. Ns, indicates results that are not statistically different compared to infected Cre^−^ *Ifnar* ^f/f^ mice.

We hypothesized that *Ifnar^−^* ^/−^ myeloid cells produced the high levels of proinflammatory cytokines in response to extensive viral replication and RNA pathogen-associated molecular pattern (PAMP) generation. To demonstrate that CD11c^+^ cells *in vivo* induced pro-inflammatory cytokines, we performed qRT-PCR analysis of CD11c^+^ cells isolated by antibody-coated magnetic bead separation from the spleens of *Ifnar* ^−/−^ mice; these cells had higher levels of IL-1ß, IL-6 and TNF-α mRNA and viral RNA compared to those isolated from Cre^−^ *Ifnar* ^f/f^ mice (**Fig. 5B**).

### MAVS-dependent induction of “cytokine storm”

WNV-infected *Ifnar* ^−/−^ CD11c^+^ cells produced proinflammatory cytokines, which suggests a linkage between uncontrolled virus replication and “cytokine storm”. A recent study suggested that in the context of LPS-priming, MAVS provides a second signal for inflammasome assembly and conversion of pro- to mature IL1-ß [30]. Consistent with our hypothesis that excessive levels of viral RNA in *Ifnar^−/−^* myeloid cells results in MAVS signaling and inflammasome activation, WNV-infected *Mavs^−/−^*×*Ifnar* ^−/−^ double knockout (DKO) mice succumbed at a later time point compared to *Ifnar* ^−/−^ mice (4.3 days compared to 2.7 days, [13]) despite the absence of pathways that both sense and control WNV infection [13], [31]–[37]. Based on these results, we predicted that the absence of MAVS signaling in WNV-infected *Mavs^−/−^*×*Ifnar* ^−/−^ DKO mice would limit cytokine induction despite high levels of infection and viral RNA generation in permissive myeloid cells.

**Figure 6:**
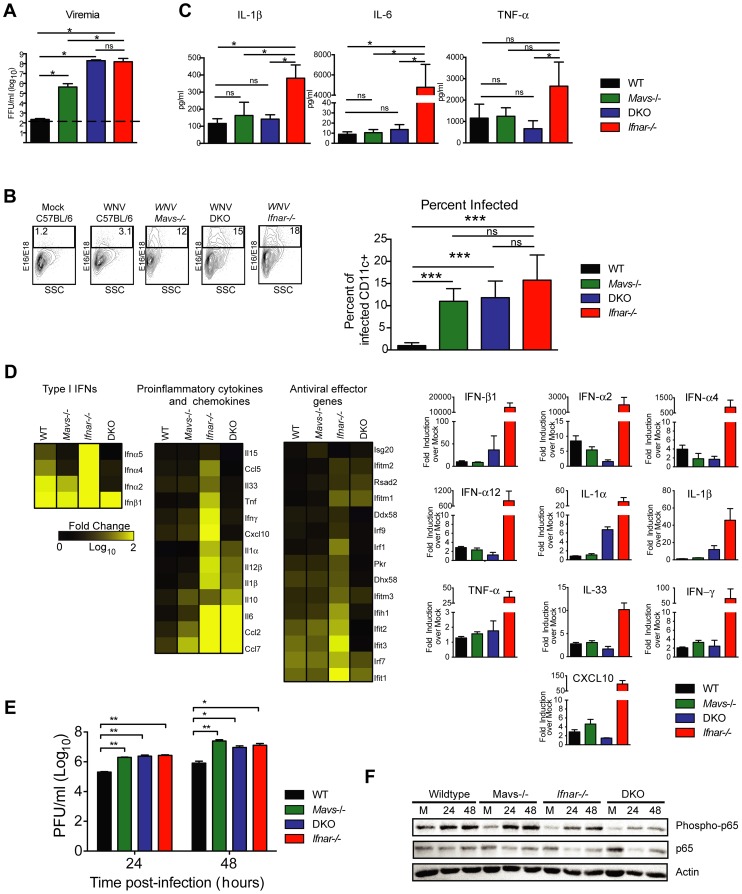
Effect of MAVS signaling on cytokine induction. **A–C**. WT, *Ifnar* ^−/−^, *Mavs* ^−/−^, or *Mavs* ^−/−^×*Ifnar* ^−/−^ DKO mice (*n* = 4 for each group) were infected with 10^2^ PFU of WNV and 48 hours later serum was collected. **A**. Viremia was determined using focus-forming assays. Data are shown as FFU per ml of serum. The dotted line represents the limit of sensitivity of the assay. Error bars indicate the SD. **B**. At 48 hours after WNV inoculation, splenocytes were gated on CD19^−^CD11c^+^CD11b^lo^ followed by intracellular staining for WNV antigen. **C**. The concentration of IL-1ß, IL-6 and TNF-α in serum was determined by cytokine bioplex assay. Mean values ± SD are shown. The IL-1ß, IL-6 and TNF-α cytokine levels were equal between *Mavs* ^−/−^, *Mavs* ^−/−^×*Ifnar* ^−/−^ DKO, and WT C57BL/6 control mice. **D**. A microarray was performed on RNA isolated from spleen of mock-infected mice (n = 2) and WNV-infected WT (n = 3), *Mavs* ^−/−^ (n = 3), *Ifnar* ^−/−^ (n = 3), and *Mavs* ^−/−^×*Ifnar* ^−/−^ DKO (n = 3) mice. A student's *t*-test (*P*≤0.01) was performed to determine the genes that had different expression levels with infection compared to levels in mock infections for each of the four mouse strains (1.5 fold change cut-off). Quantitative analysis of pro-inflammatory cytokines and chemokines is shown on the right. **E**. WT, *Ifnar* ^−/−^, *Mavs* ^−/−^, *Ifnar* ^−/−^ *×Mavs* ^−/−^ DKO BMDCs were infected with WNV and 0, 24 or 48 hours later viral burden in the supernatant was measured. The data are the mean of three independent experiments. Error bars indicate SD. Asterisks denote statistical significance relative to WT cells (*, *P*<0.05; **, *P*<0.01). **F**. Western blot showing phospho-p65 and total (relative) p65 staining in WT, *Mavs* ^−/−^, *Ifnar* ^−/−^, *Ifnar* ^−/−^ *×Mavs* ^−/−^ DKO BMDCs at 0 (M, mock), 24 or 48 after WNV infection. ß-actin staining is included as a loading control. The results are representative of two independent experiments.

To test this hypothesis, we infected *Ifnar* ^−/−^, *Mavs^−/−^*, *Mavs^−/−^*×*Ifnar* ^−/−^ DKO, and WT mice with WNV and harvested serum at 48 hours. Analysis of viremia revealed ∼10^3^ to 10^6^-fold higher levels in *Ifnar* ^−/−^ (1.6×10^8^ FFU/ml, *P* = 0.03), *Mavs* ^−/−^ (4.4×10^5^ FFU/ml, *P* = 0.03), and *Mavs^−/−^*×*Ifnar* ^−/−^ DKO (2.0×10^8^ FFU/ml, *P* = 0.03) mice compared to WT (2.3×10^2^ FFU/ml) mice (**Fig. 6A**). Among CD11c^+^ cells isolated from the spleen, a similar percentage of cells were infected with WNV in *Ifnar* ^−/−^, *Mavs^−/−^*, and *Mavs^−/−^*×*Ifnar* ^−/−^ DKO mice compared to WT mice (**Fig. 6B**). We next measured cytokine levels at 48 hours after WNV infection of *Ifnar* ^−/−^, *Mavs^−/−^*, *Mavs^−/−^*×*Ifnar* ^−/−^ DKO, and WT mice (**Fig. 6C** and **Table 2**). Only serum from the *Ifnar* ^−/−^ mice revealed the profile of elevated cytokine levels. Microarray analysis from the spleens of WNV-infected *Ifnar* ^−/−^, *Mavs^−/−^*, *Mavs^−/−^*×*Ifnar* ^−/−^ DKO, and WT mice (**Fig. 6D**) corroborated these findings. mRNA levels of cytokines associated with NF-κB and inflammasome activation (e.g., TNF-α, IL-6, IL-1ß, and IL-33) were elevated in the spleens of *Ifnar* ^−/−^ mice compared to *Mavs^−/−^*, *Mavs^−/−^*×*Ifnar^−/−^* DKO, or WT mice. To further define the linkage between MAVS and cytokine production, we infected bone marrow derived DCs (BMDCs) from *Ifnar* ^−/−^, *Mavs^−/−^*, *Mavs^−/−^*×*Ifnar* ^−/−^ DKO, and WT mice. As anticipated, higher levels of WNV infection were observed in *Ifnar* ^−/−^, *Mavs^−/−^*, *Mavs^−/−^*×*Ifnar* ^−/−^ DKO cells compared to WT cells (**Fig. 6E**). At 24 and 48 hours, we detected lower amounts of phosphorylation of the p65 subunit NF-κB in *Ifnar* ^−/−^ BMDC (**Fig. 6F**). A deficiency of MAVS alone, however, did not reduce p65 phosphorylation substantially indicating the existence of MAVS-dependent and -independent (yet IFNAR-dependent) pathways for NF-κB activation after WNV infection. In the absence of both MAVS and IFNAR, there was noticeably reduced p65 phosphorylation, as well as lower levels of IL-1ß, IL-6, CCL5, and other ISGs (IFIT1, IFIT2, and IFIT3) mRNA (**Fig. S2**); these results in BMDCs were consistent with the decrease in NF-κB-dependent cytokines produced in *Mavs^−/−^*×*Ifnar^−/−^* DKO compared to *Ifnar^−/−^* mice. The elevated expression of IFIT genes in WNV-infected *Ifnar* ^−/−^ mice was not unexpected; even though IFIT genes are ISGs, their expression can be induced directly by IRF-3 [38], [39]. In *Ifnar* ^−/−^ BMDCs, the increase in WNV infection results in enhanced RNA PAMP generation, MAVS signaling, and IRF-3 nuclear translocation, which induces IFIT genes independently of the IFN signaling pathway.

**Table 2:** Cytokine and chemokine levels in serum of WNV-infected WT, *Mavs* ^−/−^, *Ifnar* ^−/−^, and *Mavs* ^−/−^×*Ifnar* ^−/−^ DKO mice.

	WT		*Mavs* ^−/−^			*Ifnar^−/−^*			*Mavs^−/−^*×*Ifnar^−/−^* DKO		
Cytokine	pg/ml	SD	pg/ml	SD	P value	pg/ml	SD	P value	pg/ml	SD	P value
IL-1α	12.8	3.64	15.7	0.97	ns	54.9	3.05	0.029	13.13	3.42	ns
IL-1β	117	16.2	163	38.8	ns	381	38.8	0.029	142	12.6	ns
IL-2	15.2	4.55	23.04	5.93	ns	36.3	1.89	0.029	14.8	1.43	ns
IL-3	9.58	2.46	10.8	0.69	ns	15.5	0.26	0.029	9.25	1.24	ns
IL-4	ND		ND			16.9	3.53		ND		
IL-5	22.3	5.77	23.7	2.30	ns	61.8	4.42	0.029	21.6	1.43	ns
IL-6	8.97	1.39	10.5	1.56	ns	4780	1128	0.029	13.6	2.44	ns
IL-9	46.4	17.4	11.7	0.00	ns	287	44.7	0.029	11.7	0.00	ns
IL-10	31.5	13.4	32.1	11.7	ns	115	5.13	0.029	4.70	0.00	ns
IL-12p40	332	57.7	412	33.1	ns	2211	594	0.029	519	158	ns
IL-12p70	25.7	12.4	54.5	3.80	ns	316	30.6	0.029	44.8	15.6	ns
IL-13	73.8	8.16	75.9	4.84	ns	162	16.7	0.029	77.7	15.7	ns
IL-17	38.2	3.48	71.5	12.0	ns	86.1	12.0	0.029	50.8	7.26	ns
Eotaxin	37.3	10.2	52.5	11.2	ns	868	141	0.029	45.3	7.33	ns
g-CSF	48.3	2.31	129	4.75	ns	19104	4844	0.029	282	122	ns
GM-CSF	61.3	17.4	79.8	8.53	ns	361	26.3	0.029	61.7	8.56	ns
IFN-γ	2.45	1.52	3.65	0.66	ns	495	79.6	0.029	23.2	6.25	ns
KC	75.1	15.7	114	11.1	ns	5627	851	0.029	183	68.9	ns
MCP-1	175	8.78	279	14.2	ns	12743	1143	0.029	402	168	ns
MIP-1α	16.9	5.88	15.7	1.25	ns	71.5	7.85	0.029	9.86	2.42	ns
MIP-1β	18.1	1.26	32.2	5.04	ns	472	34.2	0.029	19.3	6.52	ns
RANTES	50.2	17.2	71.6	12.6	ns	405	45.9	0.029	69.7	24.3	ns
TNF-α	1156	376	1245	198	ns	2636	562	ns	661	188	ns

WT, *Ifnar* ^−/−^, *Mavs* ^−/−^, *Ifnar* ^−/−^ *×Mavs* ^−/−^ DKO (n = 4 for each group) were infected with 10^2^ PFU of WNV. After 48 hours, serum was collected and the concentration of cytokines was determined. Mean values, SD, and *P* values are compared to WT mice. Not detected (ND) indicates samples that were below the limit of detection for the assay. ns, indicates results that are not statistically different from WT mice.

### Inflammasome activation in *Ifnar* ^−/−^ CD11c^+^ cells after WNV infection

We observed higher levels of IL-1ß in the serum of *Ifnar* ^−/−^ mice, which were absent in *Mavs^−/−^*×*Ifnar* ^−/−^ DKO mice (**Fig. 6B**). This observation suggested that PRR signaling through MAVS was required for inflammasome activation. Prior studies had established that NLRP3 is the key Nod-like receptor inducing IL-1β and restricting WNV infection in mice [17]. To test whether the increase in IL-1β levels observed in WNV-infected *Ifnar* ^−/−^ mice was due to excess triggering of the NLRP3 inflammasome, we used a MAb (MAR1-5A3) to block IFNAR signaling in WT or *Nlrp3* ^−/−^ BMDCs and mice. At 48 hours after WNV infection, Western blot analysis revealed reduced amounts of cleaved IL-1ß in the MAR1-5A3-treated *Nlrp3* ^−/−^ compared to MAR1-5A3 treated-WT BMDC (**Fig. S3A**) despite equivalent levels of viral replication (**Fig. S3B**). Consistent with this, blockade of type I IFN signaling resulted in enhanced mRNA expression of IL1ß (and other cytokines, IL-6, and TNF-α) in WT but not *Nlrp3* ^−/−^ BMDCs infected with WNV (**Fig. S3C**). *In vivo*, serum cytokine levels were higher in WNV-infected WT and *Nlrp3* ^−/−^ mice treated with MAR1-5A3 compared to those receiving the isotype control MAb (**Fig. S3D**). However, with the exception of IL-1ß and possibly IL-6, cytokine levels in WNV-infected MAR1-5A3 treated *Nlrp3* ^−/−^ and WT mice were similar (**Fig. S3D** and data not shown). Although inflammasome activation generates vasoactive cytokines, in the context of the enhanced WNV replication in *Ifnar*-signaling deficient animals, this did not contribute to visceral organ injury. Consistent with this, inflammasome assembly or signaling deficient animals (*Nlpr3* ^−/−^, *IL1r* ^−/−^, or *caspase-1/11* ^−/−^ mice) that were treated with MAR1-5A3 sustained similar gross and microscopic liver and renal injury compared to WT mice treated with MAR1-5A3 or *Ifnar* ^−/−^ mice (**Fig. S3E–G**, and data not shown).

### TNF-α blockade prolongs survival after WNV infection in CD11c Cre^+^ *Ifnar* ^f/f^ mice

**Figure 7:**
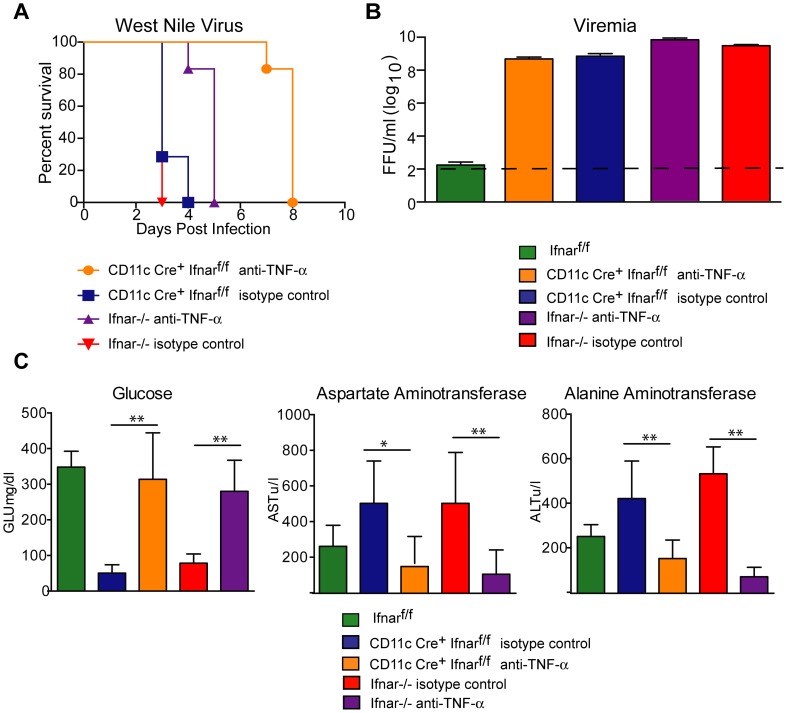
TNF-α blockade prolongs survival of WNV infected CD11c Cre^+^ *Ifnar* ^f/f^ and *Ifnar* ^−/−^ mice. **A**. Eight- to ten-week-old CD11c Cre^+^ *Ifnar* ^f/f^ and *Ifnar* ^−/−^ mice were injected via an intraperitoneal route with 200 µg of anti-TNF-α or isotype control MAbs one day prior to infection with 10^2^ PFU of WNV by footpad injection. Survival differences were statistically significant between anti-TNF-α and isotype control MAb-treated CD11c Cre^+^ *Ifnar* ^f/f^ mice (***, *P*<0.0001) and anti-TNF-α and isotype control MAb-treated *Ifnar* ^−/−^ mice (***, *P*<0.0001). The data is pooled from two independent experiments (*n* = 8 for all groups). **B**. Viral burden in serum. Eight- to ten-week-old CD11c Cre^+^ *Ifnar* ^f/f^ and *Ifnar* ^−/−^ mice were injected with 200 µg of anti-TNF-α or isotype control MAb and Cre^−^ *Ifnar* ^f/f^ were left untreated one day prior to infection with 10^2^ PFU of WNV. Infectious virus in the serum was determined from samples harvested at 48 hours post-infection. Differences were not statistically different. **C**. Eight- to ten-week-old CD11c Cre^+^ *Ifnar* ^f/f^ and *Ifnar* ^−/−^ mice were administered 200 µg of anti-TNF-α or isotype control MAb via an intraperitoneal route one day prior to inoculation of 10^2^ PFU of WNV by footpad injection. Glucose (GLU), AST, and ALT levels were analyzed from serum obtained 48 hours after infection. The data are pooled from two independent experiments (*n* = 8 for all groups). Asterisks indicate differences that are statistically significant (*, *P*<0.05; **, *P*<0.01).

To assess whether the inflammatory response contributed to the multi-organ injury and lethality, we neutralized TNF-α activity (**Fig. 7A**). Administration of a TNF-α blocking MAb (200 µg), which inhibited both soluble and cell surface-associated forms [40], one day prior to infection increased the median survival time of *Ifnar* ^−/−^ (from 3 to 5 days, *P* = 0.002) and CD11c Cre^+^ *Ifnar* ^f/f^ (from 3 to 8 days, *P* = 0.0009) mice. To support of our hypothesis that the multi-organ failure induced by “cytokine storm” contributed to disease in the *Ifnar* ^−/−^ and CD11c Cre^+^ *Ifnar* ^f/f^ mice, we measured viral titers in the serum of anti-TNF-α and isotype control MAb treated mice (**Fig. 7B**). Viremia at 48 hours post infection was not different suggesting that the beneficial effect of anti-TNF-α therapy was not due to effects on viral replication. Analysis of blood chemistries at 48 hours after infection of the CD11c Cre^+^ *Ifnar* ^f/f^ and *Ifnar* ^−/−^ mice following anti-TNF-α treatment revealed improved AST, ALT, and glucose levels that were not different from WNV-infected Cre^−^ *Ifnar* ^f/f^ control mice (**Fig. 7C** **and** **Table 3**). Cytokine analysis at 48 hours after infection showed reduced serum levels of 13 cytokines following anti-TNF-α MAb treatment in both the *Ifnar* ^−/−^ and CD11c Cre^+^ *Ifnar* ^f/f^ mice (**Table 4**). Although reduced levels of IL-1ß and IL-6 were observed in *Ifnar* ^−/−^ mice after anti-TNF-α MAb treatment, five cytokines (IL-2, IL-12p40, GM-CSF, Rantes (CCL5), and MIP1-ß (CCL4)) that were decreased in CD11c Cre^+^ *Ifnar* ^f/f^ mice were not altered in *Ifnar* ^−/−^ mice. Thus, TNF-α blockade can reduce induction of most but not all of the proinflammatory cytokines, and this correlated with Cre^+^ *Ifnar* ^f/f^ and *Ifnar* ^−/−^ animals succumbing to WNV infection less rapidly (**Fig. 7A**).

**Table 3:** Blood chemistry in MAb treated WNV-infected CD11c Cre^+^ *Ifnar* ^f/f^ and *Ifnar* ^−/−^ mice.

	AST			ALT			Glucose		
	Conc. (u/L)	SD	P value	Conc. (u/L)	SD	P value	Conc. (mg/dL)	SD	P value
**CD11c Cre^+^ anti-TNFα**	199	91.1	0.032	194	93.6	0.004	314	130	0.008
**CD11c Cre^+^ isotype**	505	238		4422	913		50.8	23.4	
***Ifnar*** **^−/−^ anti-TNFα**	83.7	43.8	0.001	208	89.2	0.001	280	87.2	0.001
***Ifnar*** **^−/−^ isotype**	6317	1320		5542	1192		82	25.7	

CD11c Cre^+^ *Ifnar* ^f/f^ and *Ifnar* ^−/−^ mice were treated with either anti-TNF-α blocking or isotype control MAbs (200 µg/mouse) one day prior to infection (*n* = 6 for each group). The mice were infected with 10^2^ PFU of WNV. At 48 hours, serum was collected and AST, ALT and glucose levels were measured. Mean values ± SD are shown. *P* values are compared to isotype control treated samples for each group. Data are pooled from two independent experiments.

**Table 4:** Cytokine levels in serum of *Ifnar* ^−/−^ and CD11c Cre^+^ *Ifnar* ^f/f^ mice after treatment with anti-TNF-α MAbs and WNV infection.

CD11c Cre *ifnar^f/f^*+isotype MAb			CD11c Cre *ifnar^f/f^*+anti-TNF-α MAb			*Ifnar^−/−^*+isotype MAb		*Ifnar^−/−^*+anti-TNF-α MAb		
Cytokine	pg/ml	SD	pg/ml	SD	P value	pg/ml	SD	pg/ml	SD	P value
IL-1α	105.2	16.37	27.54	24.47	0.0121	132.9	28.06	48.79	27.63	0.0005
IL-1β	450.9	105.8	192.1	32.23	0.0238	773.6	223.7	163	72.89	0.029
IL-2	24.53	26.98	4.246	2.015	0.0064	41.68	43.16	20.66	21.26	ns
IL-3	15.14	5.938	5.445	4.047	0.0023	28.53	12.35	8.102	7.238	0.003
IL-4	ND		ND			ND		ND		
IL-5	113.1	57.82	220.6	329.3	ns	125.9	120.3	91.73	117.1	ns
IL-6	1852	1567	96.48	91.76	0.0002	13253	5772	447.4	289	0.0002
IL-9	ND		ND			ND		ND		
IL-10	416.3	172.1	70.3	58.5	0.0013	235.8	78.57	69.05	45.95	0.001
IL-12p40	4254	1284	1162	619.7	0.0003	7544	2620	7838	2539	ns
IL-12p70	255.3	285.3	28.02	14.58	0.0007	791.7	682	172.8	177.4	0.0426
IL-13	122.9	14.82	51.44	36.08	0.0079	270.7	213	58.37	24.89	0.0061
IL-17	53.38	27.99	41.7	25.3	ns	58.27	31.21	37.4	13.52	ns
Eotaxin	1673	1128	171.7	181	0.0079	2604	949.2	351.2	218.1	0.0095
g-CSF	76450	23739	6272	12199	0.0002	95226	25232	18248	13599	0.0002
GM-CSF	455.6	365	110.8	72.58	0.0011	815.8	692	316.8	316.6	ns
IFN-γ	405.6	260.9	35.16	18.58	0.0002	331.9	132.2	131	142.9	0.028
KC	6449	2491	1022	435.7	0.0007	18053	11096	4760	2425	0.0002
MCP-1	42461	10809	5514	2526	0.0012	24715	14364	7493	7342	0.036
MIP-1α	196.6	49.52	53.02	30.35	0.0007	193.9	76.11	105.1	50.55	0.0225
MIP-1β	594.8	320.9	253.3	173.2	0.0426	698.8	587.1	445.1	206.5	ns
RANTES	974.5	754.5	163.6	87.58	0.0007	1245	1156	526.3	616.2	ns

CD11c Cre^+^ *Ifnar* ^f/f^ and *Ifnar* ^−/−^ mice were treated with either anti-TNF-α blocking or isotype MAb (200 µg/mouse) one day prior to infection (*n* = 8 for each group). The mice were infected with 10^2^ PFU of WNV and at 48 hours serum was collected and the concentration of cytokines was determined. Mean values ± SD are shown. *P* values are compared to the isotype control MAb treated samples for each group. Data are pooled from three independent experiments. ns indicates results that are not statistically different compared to mice treated with isotype control MAb. Not detected (ND) indicates samples that were below the limit of detection for the assay.

### Alternative pathway of complement activation contributes to disease pathogenesis

**Figure 8:**
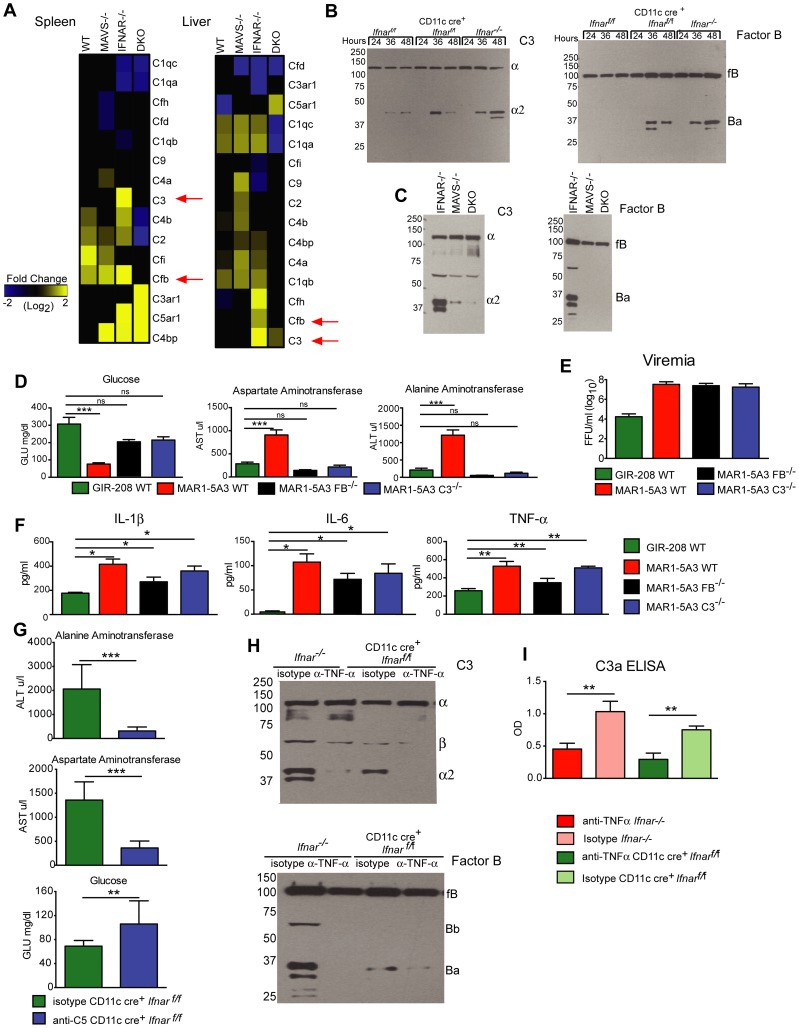
Complement activation contributes to liver injury after WNV infection. **A**. Microarray analysis of complement genes was performed on RNA isolated from spleen and liver of WNV-infected WT, *Mavs* ^−/−^, *Ifnar* ^−/−^, and *Mavs* ^−/−^×*Ifnar* ^−/−^ DKO mice. Genes that showed statistically significant increases (*P*<0.05) compared to WT are colored in yellow. Red arrows denote C3 and factor B relative mRNA levels. **B–C**. Analysis of C3 (*left*) and factor B (*right*) levels and split-products (labeled as C3-α2 and Ba) in the serum of Cre^−^ *Ifnar* ^f/f^, CD11c+ Cre^+^ *Ifnar* ^f/f^, and *Ifnar* ^−/−^ mice at 24, 36, and 48 hours after WNV infection (**B**) as determined by Western blotting. Similar experiments were performed on serum samples at 48 hours after WNV infection from *Ifnar* ^−/−^, *Mavs* ^−/−^, and *Mavs* ^−/−^×*Ifnar* ^−/−^ DKO mice (**C**). The results are representative of samples from different mice. **D**. Serum levels of glucose, AST, and ALT in WT, *C3* ^−/−^ and *factor B* ^−/−^ mice after treatment with the IFNAR blocking MAb (MAR1-5A3) and infection with WNV. The serum was harvested 72 hours after infection. For the WT mice, a comparison is made with treatment with the isotype control MAb (GIR-208). The results are the average of two independent experiments with a total of 8 mice per group, and asterisks indicate statistically significant differences (***, *P*<0.0001). **E–F**. Viremia and serum cytokines (IL-1ß, IL-6, and TNF-α) at 72 hours in WT, *C3* ^−/−^ and *factor B* ^−/−^ mice after treatment with the IFNAR blocking MAb (MAR1-5A3) and infection with WNV. For the WT mice, a comparison is made with treatment with an isotype control MAb (GIR-208) (*, *P*<0.05). **G**. Effect of treatment with C5 blocking MAb on liver injury in WNV-infected CD11c Cre^+^ *Ifnar* ^f/f^. CD11c Cre^+^ *Ifnar* ^f/f^ mice were treated with 1.25 mg (50 mg/kg) of BB5.1 anti-C5 antibody or isotype control (GIR-208) (at days −1 and +2) and infected with 10^2^ PFU of WNV on day 0. At 48 hours, serum was harvested and glucose, ALT, and AST were measured. The results are the mean of two independent experiments with n = 7 or 8 mice in total (**, *P*<0.01; ***, *P*<0.001). **H**. Western blotting analysis of C3 (left) and factor B (right) split-products (labeled as C3-α2 and Ba) in the serum of CD11c^+^ Cre^+^ *Ifnar* ^f/f^ and *Ifnar* ^−/−^ mice at 48 hours after WNV infection in animals treated with isotype or anti-TNF-α MAbs. The results are representative of two independent experiments. **I**. ELISA showing C3a levels in plasma of CD11c^+^ Cre^+^ *Ifnar* ^f/f^ and *Ifnar* ^−/−^ mice at 48 hours after WNV infection in animals treated with isotype or anti-TNF-α MAbs. The results are the mean of two independent experiments with a total of n = 6 mice and asterisks indicate significant differences (**, *P*<0.01).

Humans infected with Dengue virus, a closely related flavivirus, develop vascular leakage and sepsis syndrome and have evidence of extensive complement activation in their plasma and tissues [41]–[43]. Prior studies also have established that TNF-α regulates expression of complement genes in myeloid cells [44]. We hypothesized that complement activation and production of anaphylatoxins (e.g., C3a and C5a) might be induced by TNF-α and contribute to disease pathogenesis in WNV-infected CD11c-Cre^+^ *Ifnar* ^f/f^ mice. To evaluate this hypothesis, we first assessed mRNA levels of individual complement components in the spleen and liver of WNV-infected WT, *Mavs* ^−/−^, *Ifnar* ^−/−^, and *Mavs* ^−/−^×*Ifnar* ^−/−^ DKO mice. Notably, C3 and factor B (fB) levels were greater in WNV-infected *Ifnar* ^−/−^ mice compared to the other genotypes (**Fig. 8A**) suggesting that expression of key complement components was regulated via a MAVS-dependent pathway. We next examined complement activation in serum by assessing levels of complement proteins and split products by Western blot and ELISA. At 24 to 48 hours after WNV infection, levels of C3, fB, and their cleaved products of (C3-α2 and Ba) were higher in *Ifnar* ^−/−^ and CD11c-Cre^+^ *Ifnar* ^f/f^ mice compared to WT mice (**Fig. 8B**). These results confirm the induction of key complement proteins and activation of the complement cascade *in vivo*. In contrast, C3 and fB cleavage products were decreased or absent in the serum of WNV-infected *Mavs* ^−/−^ or *Ifnar* ^−/−^×*Mavs* ^−/−^ DKO mice at 48 hours (**Fig. 8C**), indicating that RLR signaling through MAVS was required for complement induction and activation. Consistent with a role for complement contributing to pathogenesis, liver injury at 48 hours was minimized in WNV-infected *C3* ^−/−^ and *fB* ^−/−^ mice given the IFNAR-blocking MAb MAR1-5A3 compared to similarly-treated WT mice. Analysis of blood chemistries after WNV infection of MAR1-5A3 treated *C3* ^−/−^ and *fB* ^−/−^ mice revealed normal AST and ALT values, compared to MAR1-5A3 treated C57BL/6 control mice (**Fig. 8D**), even though viremia and cytokine levels remained elevated (**Fig. 8E and F**). While liver injury persisted in MAR1-5A3-treated *Mbl-a* ^−/−^×*Mbl-c* ^−/−^ DKO or *C4* ^−/−^ mice, indicating that disease was induced primarily by the alterative and not classical or lectin pathways of complement activation, glycemia was restored to normal levels. A partial phenotype also was observed in MAR1-5A3-treated *C3aR* ^−/−^ mice, which delete the receptor for the complement anaphylatoxin C3a: Cytokine (IL-1ß, IL-6, and TNF-α) and liver enzymes levels remained elevated in the serum but glucose levels normalized (**Fig. S4**). In comparison, treatment of CD11c-Cre^+^ *Ifnar* ^f/f^ mice with neutralizing MAbs against C5 reduced liver injury and prevented hypoglycemia compared to animals administered isotype control MAbs (**Fig. 8G**). To link our findings showing TNF-α signaling and complement activation contribute to WNV-induced disease and sepsis, we assessed complement activation in WNV-infected CD11c-Cre^+^ *Ifnar* ^f/f^ mice treated with blocking anti-TNF-α MAbs. Lower levels of C3 and factor B split-products (C3-α2, C3a, and Ba) were observed in the serum and plasma of mice administered blocking TNF-α compared to isotype control MAbs (**Fig. 8H and I**). These experiments place pathologic complement activation downstream of viral infection, MAVS signaling, and TNF-α signaling and indicate that it contributes to the sepsis-like syndrome.

## Discussion

Although the contribution of type I IFN signaling to the control of viral infection *in vivo* is established, its importance in specific cell subsets remains less well understood. We evaluated the function of type I IFN signaling in myeloid cells *in vivo* against WNV infection. We targeted these cells because prior studies had indicated that WNV replicated preferentially in CD11b^+^ and CD11c^+^ cells [12], [45]. A striking phenotype was observed after WNV infection of CD11c or LysM Cre^+^ *Ifnar* ^f/f^ mice, as these animals succumbed with a similar rate and kinetics compared to the complete gene deletion *Ifnar* ^−/−^ mice. In contrast, deletion of IFNAR expression only on B cells (CD19 Cre^+^ *Ifnar* ^f/f^) showed a phenotype after WNV infection that resembled the Cre^−^ *Ifnar* ^f/f^ mice. Viral burden in several organs of CD11c Cre^+^ *Ifnar* ^f/f^ mice was markedly elevated and approached that seen in *Ifnar* ^−/−^ mice. Our experiments revealed a dominant antiviral effect *in vivo* of type I IFN signaling in myeloid cells, either CD11c^+^ cells or MØ/monocytes/granulocytes. Pathological analyses showed preferential WNV infection of *Ifnar* ^−/−^ myeloid cells, and this resulted in “cytokine storm”, which was associated with liver and kidney damage, and rapid death. High levels of WNV RNA in *Ifnar* ^−/−^ myeloid cells triggered uncontrolled production of proinflammatory cytokines, pathological complement induction and activation, which together caused a sepsis-like syndrome. This phenotype did not occur universally: whereas 100% of *Ifnar* ^−/−^ mice succumbed to infection with the arthritogenic CHIKV alphavirus, no disease or lethality was observed in CD11c or LysM Cre^+^ *Ifnar* ^f/f^ mice.

The phenotype after WNV infection of CD11c or LysM Cre^+^ *Ifnar* ^f/f^ mice varies from that observed after infection with MHV [20] or MNoV [22]. In the MHV study, increased vulnerability was observed in LysM Cre^+^ *Ifnar* ^f/f^ mice but it did not recapitulate that seen in *Ifnar* ^−/−^ mice. In comparison, deletion on *Ifnar* on CD11c^+^ cells resulted in only a subset (∼30%) of animals dying, and this occurred almost a week later. In the MNoV study, IFNAR-dependent responses in MØ and DCs limited viral infection but were dispensable for preventing lethal infection, as all MNoV-infected CD11c or LysM Cre^+^ *Ifnar* ^f/f^ animals survived in contrast to *Ifnar* ^−/−^ mice. Systemic cytokine levels were not measured in either study. Related to these findings, cell type-restricted deletion of *Stat1* in MØ, monocytes, and granulocytes (LysM Cre^+^ *Stat1* ^f/f^) but not DCs (CD11c Cre^+^ *Stat1* ^f/f^) resulted in enhanced lethality after *Listeria monocytogenes* infection [20], [21]. Higher levels of cytokines and chemokines were observed in *Listeria*-infected LysM Cre^+^ *Stat1* ^f/f^ mice, although they were lower than in infected *Stat1* ^−/−^ mice. Analogous to our studies, *Listeria*-infected mice lacking IFNAR expression on MØ also sustained liver damage. Thus, loss of IFNAR expression in distinct populations of cells can enhance pathogenesis of viral and bacterial pathogens, although the level of mortality and cytokine production can vary.

Our IHC and flow cytometric analysis of tissues and blood revealed that a demonstrable fraction of infected cells in CD11c Cre^+^ *Ifnar* ^f/f^ mice belonged to the CD11c^+^ subset. It remains unclear why only a fraction (∼5 to 10%) of CD11c^+^ cells were targeted; this could reflect a difference in vulnerability of DC subsets or a stochastic process. A similar phenomenon was observed *ex vivo* after WNV infection of *Ifnar* ^−/−^ BMDCs, in which a maximum of 10 to 20% of cells was infected even at high multiplicities of infection [35]. In comparison, CHIKV does not target myeloid cells for infection in mice [28], [46], and thus may not gain the same replication advantage in CD11c Cre^+^ *Ifnar* ^f/f^ or LysM Cre^+^ *Ifnar* ^f/f^ animals.

Prior studies in *Ifnar* ^−/−^ mice with virulent or attenuated WNV strains revealed enhanced infection and lethality [12], [32]–[34]. While the susceptibility phenotype is related to the higher levels of WNV replication, the contribution of cytokines was not assessed. Although we measured high viral titers in the serum, organs, and brains of CD11c Cre^+^ *Ifnar* ^f/f^ and *Ifnar* ^−/−^ mice, IHC revealed less viral antigen staining in the brain parenchyma than anticipated by the viral titer data. This suggested to us that (a) the high levels of WNV in brain homogenates could be due to infected cells in the intravascular space that were not removed despite extensive perfusion; and/or (b) although CD11c Cre^+^ *Ifnar* ^f/f^, LysM Cre^+^ *Ifnar* ^f/f^ and *Ifnar* ^−/−^ mice rapidly succumbed to WNV, it might not be due to infection in the brain. Indeed, high levels of TNF-α and IL-1ß and other proinflammatory cytokines were present in the serum of CD11c or LysM Cre^+^ *Ifnar* ^f/f^ and *Ifnar* ^−/−^ mice but not in infected Cre^−^ *Ifnar* ^f/f^ mice at 48 hours. Vasoactive cytokines affect blood vessel permeability and vascular tone [47], [48], and could have limited perfusion in the CD11c Cre^+^ *Ifnar* ^f/f^ and *Ifnar* ^−/−^ mice. Our results are most consistent with a model in which cytokine-mediated changes to the vasculature limited organ perfusion and resulted in high titers that reflect viral burden in blood rather than replication within the parenchyma of some tissues.

“Cytokine storm” reflects excessive cytokine production that occurs following infection with gram-negative bacteria or pathogenic influenza A viruses (reviewed in [47], [48]). Excessive cytokine production with vascular permeability changes also has been described for flaviviruses in the context of severe dengue virus infection in humans or IFN-signaling-deficient mice [49]–[51]. Our studies in CD11c Cre^+^ *Ifnar* ^f/f^ and *Ifnar* ^−/−^ mice revealed that serum levels of twenty different inflammatory cytokines were elevated at 48 hours after WNV inoculation. Loss of type I IFN signaling on the CD11c^+^ or other myeloid cells affected viral tropism and resulted in dysregulated cytokine responses after WNV infection. Combining this with the likely changes in vascular resistance that affected perfusion, our results are compatible with a clinical picture of “cytokine storm”.

WNV-infected CD11c or LysM Cre^+^ *Ifnar* ^f/f^ and *Ifnar* ^−/−^ mice developed marked elevations in serum liver enzymes and profound hypoglycemia, consistent with hepatocellular injury. Histopathological analysis of the liver in CD11c Cre^+^ *Ifnar* ^f/f^ and *Ifnar* ^−/−^ mice revealed zonal coagulative necrosis with apoptotic hepatocytes. Similarly, in the spleen and LN marked changes in the organ architecture and cell viability were apparent. While viral antigen staining was present in lymphoid organs, it was limited in the liver, and suggested that end-organ damage was secondary to ischemia and/or toxic effects of cytokines rather than direct virus-induced apoptosis.

The high levels of IL-1ß in the serum of the WNV-infected CD11c Cre^+^ *Ifnar* ^f/f^, LysM Cre^+^ *Ifnar* ^f/f^ and *Ifnar* ^−/−^ mice indicated significant inflammasome activation. WNV activates the NLRP3 inflammasome to produce mature IL-1ß, which helps control infection in the brain through a pathway that synergizes with type I IFN signaling [15], [17]. Inflammasome activation requires two signals: signal 1 induces transcription of IL-1β (reviewed in [52]) and signal 2 promotes assembly of a multi-protein complex that activates caspase-1 to cleave IL-1ß and IL-18 to their mature forms [53]. The high amount of IL-1ß detected in the serum of the *Ifnar* ^−/−^ and CD11c or LysM Cre^+^ *Ifnar* ^f/f^ mice that indicates inflammasome activation during WNV infection occurs independently of IFNAR signaling in infected myeloid cells. The lower levels of cytokines in the serum of *Mavs* ^−/−^×*Ifnar* ^−/−^ DKO mice establish that inflammasome activation following WNV infection occurs in part, via a MAVS-dependent pathway.

Recent studies in the context of bacterial infection have suggested a role for IFNAR signaling in inflammasome activation [17], [31], [32], [34], [54]–[57] and production of vasocative cytokines, including IL-1ß. Whereas in the context of *Francisella tularensis* and *Listeria monocytogenes* infection IFNAR expression was required for inflammasome activation [58], in our study, MAVS and not IFNAR signaling played the dominant role. In animals lacking IFNAR expression on CD11c^+^ cells, enhanced WNV replication produces excessive viral RNA PAMPs that are recognized by the cytosolic PRR, RIG-I and MDA5 [59]–[62]. This sensing event signals through MAVS to induce proinflammatory cytokines through inflammasome-dependent (IL-1§) and -independent (e.g., NF-κB) pathways. Accordingly, the combined absence of IFNAR and MAVS resulted in enhanced infection without early “cytokine storm”, with DKO mice succumbing to infection days later likely due to massive virus infection in the central nervous system. The importance of the NF-κB pathway in cytokine induction is highlighted by WNV infection studies in *Irf3* ^−/−^×*Irf7* ^−/−^ DKO mice, which still showed evidence of liver injury (high serum AST and ALT) and over-exuberant cytokine production (A. K. Pinto and M. S. Diamond, unpublished results).

Consistent with our hypothesis that proinflammatory cytokines contributed to WNV-induced lethality in CD11c Cre^+^ *Ifnar* ^f/f^ and *Ifnar* ^−/−^ mice, administration of a blocking TNF-α MAb prolonged survival but did not affect viral replication. Moreover, pre-treatment with anti-TNF-α MAbs resulted in lower systemic cytokine levels and less end-organ tissue injury at 48 hours after infection. Somewhat surprisingly, disruption of inflammasome activation in *Nlrp3* ^−/−^, *IL1r* ^−/−^, or *caspase-1/11* ^−/−^ mice did not affect WNV-induced liver and renal injury when IFNAR signaling was blocked. Analogous results were observed in *Ifnar* ^−/−^ mice treated with neutralizing anti-IL-1 ß and IL-6 MAbs (A. K. Pinto and M. S. Diamond, unpublished results). Thus, even though there was marked inflammasome activation in the context of “cytokine storm”, these cytokines were not primarily responsible for the severe clinical phenotype.

**Figure 9:**
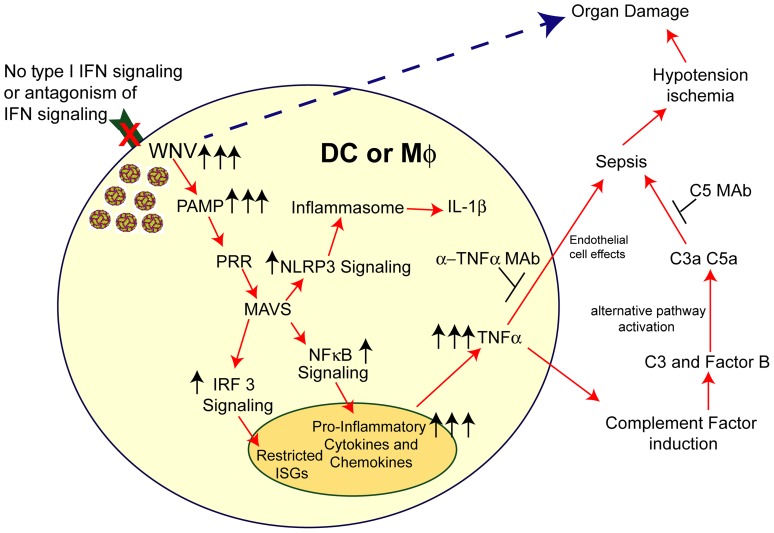
Model of sepsis after viral infection of CD11c^+^ DC or LysM^+^ myeloid cells. Myeloid cells that lack signaling (due to *Ifnar* gene deletion, pharmacological blockade with anti-IFNAR MAbs, or IFN signaling antagonism by viruses) are more susceptible to infection. In the context of WNV infection, increased viral replication results in enhanced RNA PAMP generation, which activates MAVS via recruitment to the mitochondria. This results in downstream activation of IRF-3 and NF-κB and also assembly of the NLRP3 inflammasome. IRF-3 activation and nuclear translocation promotes induction of a limited set of ISGs. NF-κB activation and nuclear translocation promotes expression of pro-inflammatory cytokines (e.g., IL-6 and TNF-α) and chemokines (CCL5 and CXCL10). Soluble TNF-α can modulate endothelial cells function and integrity and also induce complement factor expression in myeloid and hepatic cells, including C3 and factor B. Higher levels of C3 and factor B in the context of increased WNV in plasma results in excessive complement activation (via the alternative pathway), which can liberate the C3a and C5a anaphylatoxins. Along with TNF-α, these promote changes to vascular permeability and tone that result in hypotension and visceral organ (e.g., liver and kidney) damage. Independently, excessive virus infection in restricted tissues (e.g., spleen and LN) can cause organ damage. The pathological effects of this cascade can be mitigated by administration of blocking MAbs to TNF-α or C5.

So how did TNF-α promote the sepsis-like syndrome associated with enhanced WNV infection of myeloid cells? Although high levels of serum TNF-α can have vasoactive effects that alter endothelial cell barrier integrity [63]–[65], our experiments suggest alternative pathway complement activation and possibly resultant production of C5a anaphylatoxin contributed to the phenotype. Liver injury was minimized in WNV-infected *C3* ^−/−^, *fB* ^−/−^, and C5-depleted mice lacking type I IFN signaling, indicating that tissue damage required activation of the alternative complement pathway. A survival benefit was not observed in WNV-infected *C3* ^−/−^, *fB* ^−/−^, and C5-depleted mice lacking type I IFN signaling (A. Pinto and M. Diamond, unpublished results), likely because of the rapid spread of virus to the central nervous system due to an absence of the protective antiviral effects of complement [66] and type I IFN [67]. Associated with WNV infection of CD11c Cre^+^ *Ifnar* ^f/f^ and *Ifnar* ^−/−^ mice, we observed massive activation of complement including accumulation of C3 and fB split products. The induction of C3, fB, and other complement proteins in the spleen and liver occurred after WNV infection in a MAVS-dependent manner. Indeed, in the serum of mice lacking MAVS alone or MAVS and IFNAR, we failed to observe the accumulation of C3 and fB cleavage products after WNV infection. These and other results place complement induction and/or activation downstream of TNF-α, possibly through direct actions on myeloid cells and/or hepatocytes. We propose a model for virus-induced sepsis-like syndrome (**Fig. 9**) in which infection in myeloid cells results in excessive viral RNA production, RIG-I and/or MDA5 recognition, MAVS signaling, NF-κB activation, proinflammatory cytokine production (including TNF-α), complement protein induction, alternative pathway activation, and complement anaphylatoxin production. Enhanced infection in myeloid cells could occur because of experimental blockade or genetic deficiency of IFNAR or through virus-induced immune evasion mechanisms; many human viruses target and disenable key components of the type I IFN signaling cascade [68]–[71]. As recent studies with bacterial sepsis models also suggest key roles for fB and complement peptides in mediating vascular leakage and hemodynamic instability [72]–[74], pharmacological blockade of C3a and C5a might mitigate the sepsis-like syndrome after viral infections, including the more globally relevant flavivirus, Dengue virus.

In summary, our data establishes that the selective loss of IFNAR expression on myeloid cell subsets results in an unanticipated rapid mortality following WNV infection, which was not observed when IFNAR was removed from CD19 cells or in other viral models, which still are restricted by IFNAR signaling pathways in other cells. An absence of IFNAR signaling in myeloid cells facilitated WNV infection and caused MAVS-dependent dysregulated cytokine responses that promoted induction and activation of the alternative complement pathway, which resulted in tissue injury. As different viruses antagonize IFNAR function by targeting distinct steps in the downstream signaling pathway including Jak1, Tyk2, Stat1, and Stat2 phosphorylation (reviewed in [75]), this model of infection and inflammation may be relevant in the context of other infections. Given these findings, targeted anti-cytokine and/or immunomodulatory agents may be a possible therapeutic option when sepsis is induced by massive viral infection in subsets of myeloid cells.

## Materials and Methods

### Ethics statement

This study was carried out in strict accordance with the recommendations in the Guide for the Care and Use of Laboratory Animals of the National Institutes of Health. The protocols were approved by the Institutional Animal Care and Use Committee at the Washington University School of Medicine (Assurance Number: A3381-01). Dissections and footpad injections were performed under anesthesia that was induced and maintained with ketamine hydrochloride and xylazine, and all efforts were made to minimize suffering.

### Mice

Wild type C57BL/6 mice were purchased commercially (Jackson Laboratories). *Ifnar* ^−/−^ mice were obtained from J. Sprent (Scripps Institute, San Diego CA) and backcrossed ten times onto the C57BL/6 background. CD11c Cre^+^ *Ifnar* ^f/f^, LysM Cre^+^ *Ifnar* ^f/f^, mice and *Ifnar* ^f/f^ were obtained from R. Schreiber (St. Louis, MO) and U. Kalinke (Hannover, Germany). The *Ifnar* ^f/f^ [76], CD19 Cre^+^, LysM Cre^+^, and CD11c Cre^+^ mice (Jackson Laboratories) were backcrossed using speed congenic analysis so they were 99% C57BL/6 as judged by microsatellite analysis. *Mavs^−/−^* and *Mavs^−/−^×Ifnar^−/−^* DKO mice have been described previously [13]. *Nlrp3* ^−/−^, *IL1r* ^−/−^, *caspase-1/11* ^−/−^ mice have been reported previously [42]. *C3* ^−/−^, *C3aR* ^−/−^ (gift of R. Wetsel, Houston, TX), *C4* ^−/−^, factor B^−/−^, *Mbl-a^−/−^×Mbl-c* ^−/−^ DKO mice have been reported previously [77], [78]. All mice were housed in a pathogen-free mouse facility at the Washington University School of Medicine or the University of Washington and experiments were performed in accordance and with approval of federal and University regulations. Mice (8 to 12 week-old) were inoculated subcutaneously via footpad injection with 10^2^ plaque-forming units (PFU) of WNV-NY or 10 PFU of CHIKV-LR.

### Viruses and cells

The WNV-NY strain 3000.0259 (WNV-NY) used was isolated in New York in 2000 [79] and passaged twice in C6/36 *Aedes albopictus* cells. WNV isolate, TX 2002-HC (WNV-TX02), was titered by a standard plaque assay on BHK21 cells and working stocks were generated as previously described [14]. The CHIKV strain was isolated from an infectious clone of CHIKV La Reunion 2006 OPY-1 (strain 142, CHIKV-LR, gift from S. Higgs (Manhattan, KS)) [80] and was passaged in C6/36 *Aedes albopictus* cells. BMDCs were generated as previously described [81].

### Analysis of IFNAR expression, cytokine production, and WNV infection

Forty-eight hours after WNV infection, blood was obtained by intracardiac heart puncture and spleens were recovered. Live cells were stained with MAbs specific for CD11c, CD8, CD4, Ly6G, CD11b, CD3, CD19, and IFNAR (Biolegend) to define cell types and determine IFNAR expression. To determine which leukocytes were infected with WNV, after incubating with MAbs against specific leukocyte surface markers, cells were fixed and permeabilized with Fixation and Permeabilization buffers (eBioscience), and stained with a combination of two mouse anti-WNV specific MAbs (WNV E16 and WNV E18) and a MAb against IFN-γ (Biolegend) [82]. All samples processed on an LSRII or LSRFortessa flow cytometer (BD Biosciences). The resulting data was analyzed using FlowJo (Treestar).

### Measurement of viral burden

Forty-eight hours after WNV infection, serum was obtained by intracardiac heart puncture, followed by intracardiac perfusion (20 ml of PBS), and organ recovery. Organs were weighed, homogenized using a bead-beater apparatus, and titrated by focus-forming assay [83] on Vero cells. Infected cell foci were stained with a flavivirus-cross-reactive, chimeric mouse-human MAb (WNV E18, 1 µg/ml) [84] for one hour at 37°C and then washed. Foci were detected after the cells were incubated with a 1∶2,000 dilution of horseradish peroxidase (HRP)-conjugated goat anti-human IgG (Sigma) for one hour. Staining was visualized by addition of TrueBlue detection reagent (KPL). Spots were analysis with a Biospot counter (Cellular Technology) using Immunocapture software.

### Histology, IHC, and TUNEL staining

Eight to nine-week old *Ifnar* ^−/−^, CD11c Cre^+^ *Ifnar* ^f/f^, or Cre^−^ *Ifnar* ^f/f^ mice were infected with WNV. Forty-eight hours later, mice were perfused sequentially with 20 ml PBS and 20 ml 4% PFA in PBS, and tissues were harvested and fixed in 4% PFA in PBS overnight at 4°C. Staining (hematoxylin and eosin or for WNV antigen) of paraffin-embedded tissue sections was performed as previously described [85]. After blocking non-specific binding sites, sections were incubated overnight at 4°C with an anti-WNV hyperimmune rat sera [24]. Primary antibodies were detected with secondary HRP goat anti-mouse or rat IgG (Molecular Probes). Nuclei were counter-stained with To-Pro3 (Molecular Probes). For TUNEL staining, sections were deparaffinized and rehydrated by heating to 57°C for 5 minutes then incubated with xylene, 100% ethanol, 95% ethanol, 70% ethanol and distilled water. Cells were permeabilized in proteinase K (Roche) for 30 minutes. DNase (Sigma) treatment for 10 minutes was used to introduce nicks into DNA as a positive control. *In Situ* Cell Death Detection KIT, TMR Red (Roche) was used for Tdt-mediated dUTP nick end labeling (TUNEL) and the manufacturer's protocol was followed. Cells were counter-stained with DAPI (Invitrogen) for five minutes. Slides were visualized using an Axioscope (Zeiss) microscope. Images were captured with an AxioCam HRm (Zeiss) and Axiovision Rel4.8 (Zeiss) software was used. The control and experimental images were collected and processed using the same instrument settings.

### Cytokine bioplex assay

WT, *Ifnar* ^−/−^, LysM Cre^+^ *Ifnar* ^f/f^, CD11c Cre^+^ *Ifnar* ^f/f^, Cre^−^ *Ifnar* ^f/f^, *Mavs^−/−^*, *Mavs^−/−^*×*Ifnar^−/−^*, *Mbl-a* ^−/−^×*Mbl-c* ^−/−^, *C4* ^−/−^, *C3* ^−/−^, fB^−/−^, or *C3aR* ^−/−^ mice were infected with WNV, and at specified times blood was collected and serum was isolated. The BioPlex Pro Assay was performed according to the manufacturer's protocol (BioRad). The cytokine screen included IL-1α, IL-1ß, IL-2, IL-3 IL-4, IL-5, IL-6, IL-9, IL-10, IL-12p40, IL-12p70, IL-13, IL-17, Eotaxin, G-CSF, GM-CSF, IFN-γ, KC, MCP-1 MIP-1α, MIP-1ß, RANTES (CCL5), and TNF-α.

### Blood chemistry analysis

*Ifnar* ^−/−^, LysM Cre^+^ *Ifnar* ^f/f^, CD11c Cre^+^ *Ifnar* ^f/f^, or Cre- *Ifnar* ^f/f^ mice were infected with WNV. At specified times, blood was collected by intracardiac heart puncture and serum was isolated. Prior to analysis all samples were treated with 1/2,000 dilution of ß-propiolactone (Sigma) for 30 minutes to inactivate infectious viral particles. Control experiments confirmed that β-propiolactone did not impact chemistry results (data not shown). The Diagnostic Laboratory in the Division of Comparative Medicine at Washington University performed the blood chemistry analysis.

### Complement activation analysis

To obtain mouse serum, whole blood was clotted on ice for 20 minutes before centrifugation (10,000 g×10 min at 4°C). Fresh serum (15 µl, diluted 1/100) was mixed with an equal volume of 2× SDS-PAGE Sample Buffer containing β-mercaptoethanol (Sigma). After a 4 minute incubation at room temperature (RT) the samples were heated at 65°C for 4 minutes. The samples were subjected to 10% SDS-PAGE using Tris-Glycine running buffer and then transferred to nitrocellulose membranes. The membranes were blocked overnight with 5% nonfat dried milk in Tris-borate saline (TBS) buffer. Primary goat anti-mouse C3 (1/10,000 dilution; MP Biomedicals) or goat anti-human factor B (1/5,000 dilution; Complement Technology, Inc.) antibodies were incubated with the membranes for 1.5 hours at RT. After three washes with TBS containing 0.05% Tween 20, HRP-conjugated rabbit anti-goat IgG (Sigma) was incubated for 1 h at 37°C. After three washes with TBS-Tween 20, membranes were visualized using a SuperSignal West Kit (Pierce).

### C3a ELISA

Anti-TNF-α or isotype control MAb treated CD11c Cre^+^ *Ifnar* ^f/f^ mice were infected with WNV. At 48 hours after infection, blood was collected by intracardiac heart puncture into EDTA-coated tubes. Plasma was isolated and immediately added to microtiter plates that had been coated overnight with C3a capture antibody (4 µg/ml, BD Biosciences) and blocked with PBS and 1% BSA (Sigma) for one hour. Plates were incubated for two hours at room temperature, and after washing, C3a detection antibody (0.5 µg/ml, BD Biosciences) was added for two hours at room temperature. After washing, streptavidin-HRP (Invitrogen) was added for 30 minutes at room temperature, and the plates were developed with tetramethylbenzidine substrate (Dako) and H_2_SO_4_. The adjusted OD_450_ was determined by subtracting the OD_450_ value for each sample on blocked control wells analyzed in parallel. Titers represent the serum dilution yielding an adjusted OD_450_ value equivalent to three standard deviations above the background of the assay.

### qRT-PCR

RNA was isolated from serum using an RNA isolation Kit (Qiagen) and measured by fluorogenic qRT-PCR using primers and probes (sequences available upon request) to the WNV envelope gene, IFIT1, IFIT2, IFIT3, IL-1ß, IL-6, TNF-α, and CCL5 using the One-Step RT-PCR Master Mix, and a 7500 Fast Real-Time PCR System (Applied Biosystems).

### MAb treatments

The anti-mouse TNF-α MAb CNTO-5048 and the isotype control MAb CNTO-1322 are rat/mouse chimeric monoclonal MAbs and were the generous gift of D. Shealy (Janssen R&D, Spring House, PA). The CD11c Cre^+^ *Ifnar* ^f/f^, or Cre^−^ *Ifnar* ^f/f^ mice were treated with a single intraperitoneal dose of 200 µg (∼10 mg/kg) one day prior to infection with 10^2^ PFU of WNV-NY. Anti-TNF-α and the isotype control MAb treated mice were monitored for survival or serum and organs harvested at 48 hours for further analysis. To block IFNAR signaling, WT or specific KO mice were treated with a single 1 mg (40 mg/kg) dose of MAR1-5A3 or isotype control MAb (GIR-208) one day prior to infection, as described previously [18]. To block C5 function, CD11c Cre^+^ *Ifnar* ^f/f^ mice were treated with two 1.25 mg (50 mg/kg) doses of BB5.1 or isotype control MAb (GIR-208) at days −1 and +2 relative to WNV infection, as described previously [86].

### Microarray analysis

Expression oligonucleotide arrays were performed on RNA isolated from spleen and liver tissues from strain and time-matched mock infected mice and WNV-infected WT, *Mavs^−/−^*, *Ifnar^−/−^*, and *Mavs^−/−^*×*Ifnar^−/−^* DKO mice. RNA was prepared as previously described [13]. Raw data were loaded into a custom-designed laboratory information management system (LIMS). Data were warehoused in a Labkey system (Labkey, Inc., Seattle, WA) and analyzed using GeneData Analyst 2.2.1 software (GeneData Solutions In Silico, San Francisco, CA), and TIBCO Spotfire with Integromics. Raw microarray data have been deposited in NCBI's Gene Expression Omnibus under GEO Series accession number GSE39259 and also are accessible via the Katze Laboratory website (www.viromics.washington.edu) in accordance with proposed Minimum Information About a Microarray Experiment (MIAME) standards. A student's *t*-test was performed to determine the genes that had different expression levels after WNV infection compared to mock infection for each of the four mouse strains.

### Western blotting

WT, *Mavs^−/−^*, *Ifnar^−/−^*, and *Mavs^−/−^×Ifnar^−/−^* DKO BMDCs were infected with WNV at an MOI of 2.5 and harvested and lysed 48 hours later. WT and *Nlrp3* ^−/−^ BMDC were treated for 30 minutes prior to infection with 25 µg/ml of MAR1-5A3 or GIR-208 [87] and this was maintained throughout the assay. BMDC were lysed in RIPA buffer (10 mM Tris, 150 mM NaCl, 0.02% sodium azide, 1% sodium deoxycholate, 1% Triton X-100, and 0.1% SDS, pH 7.4), with protease inhibitors (Sigma). Samples were resolved by electrophoresis on 10% SDS-polyacrylamide gels. Following transfer of proteins, membranes were blocked with 5% non-fat dried milk and probed with the following panel of primary antibodies: anti-WNV NS3 (R&D Systems), anti-mouse tubulin (Sigma), anti-mouse Ifit2 (gift of Dr. G. Sen, Cleveland, OH), anti-mouse STAT1 (Cell Signaling), anti-mouse IL-1ß (pro and cleaved forms, Abcam), anti-phospho-p65 (Ser536; Cell Signaling), and anti-ß-actin (Cell Signaling).

### Data analysis

All data was analyzed using Prism software (GraphPad4, San Diego, CA). Kaplan-Meier survival curves were analyzed by the log rank test. Differences in viral burden, cytokine levels and blood chemistries were analyzed by the Mann-Whitney test.

## Supporting Information

Figure S1 **Serum cytokine levels in CHIKV-infected mice.** *Ifnar* ^−/−^, CD11c Cre^+^ *Ifnar* ^f/f^, and Cre^−^ *Ifnar* ^f/f^ mice (n = 6 for each group) were infected with 10 PFU of CHIKV. Seventy-two hours later, serum was collected and the concentration of IL-1ß, IL-6, and TNF-α present was determined. Mean values and SD are shown. Asterisks indicate differences that are statistically significant (*, *P*<0.05).
(PDF)

Figure S2 **Levels of cytokines, ISGs, and chemokines in WT and KO DCs after WNV infection.** qRT-PCR of WT, *Mavs* ^−/−^, *Ifnar* ^−/−^, and *Mavs* ^−/−^×*Ifnar* ^−/−^ DKO DCs 24 and 48 hours after WNV infection. Relative RNA levels of IL-1ß, IL6, CCL5, IFIT1, IFIT2, and IFIT3 are shown and compared to uninfected cells. Mean values ± SD are shown. Asterisks indicate differences that are statistically significant (*, *P*<0.05; **, *P*<0.01).
(PDF)

Figure S3 **Effects of the NLRP3 inflammasome on cytokine induction.** BMDCs (*n* = 3) from C57BL/6 or *Nlrp3* ^−/−^ mice were pretreated with 25 µg/ml of the IFNAR receptor blocking antibody MAR1-5A3 or an isotype control GIR-208 for 30 minutes prior to infection with WNV and at 48 hours cells were collected. **A**. Western blot showing the expression of STAT1, the ISG IFIT2, the WNV protein NS3, and IL-1ß cleavage. **B**. Viral titers from the treated BMDCs were determined by a focus-forming assay. Data are shown as FFU per ml. Error bars indicate SD. **C**. Relative cytokine mRNA levels at 48 hours from WT or *Nlrp3* ^−/−^ BMDCs infected with WNV after treatment with MAR1-5A3 or an isotype control MAb. Gene expression was measured by qRT-PCR and normalized to *Gapdh* levels. Data is displayed as the fold increase compared to untreated cells on a log2 scale. Data represent the average of three independent experiments. Error bars indicate SD. The limit of detection was assigned as a value log2 ΔΔCt of −2. **D**. The concentration of IL-1β, IL-6 and TNF-α in serum from the treated WT and *Nlrp3^−^* ^/−^ mice was determined by cytokine bioplex assay. Mean values ± SD are shown. **E–G**. WT, *Nlrp3^−^* ^/−^, *caspase-1/11* ^−/−^ or *IL-1R* ^−/−^ mice were pretreated one day prior to WNV infection with 1 mg (40 mg/kg) of the IFNAR receptor blocking antibody MAR1-5A3 or isotype control MAb (GIR-208) prior. At 72 hours after infection, serum was collected and analyzed for ALT (**E**), AST (**F**), and glucose (**G**).
(PDF)

Figure S4 **Impact of targeted deletion of mannose binding lectins, C4, or C3a receptor on cytokine levels, liver injury, and glycemia in mice lacking IFNAR signaling and infected with WNV.** WT, *Mbl-a* ^−/−^×*Mbl-c* ^−/−^ (MBL/AC^−/−^), *C4* ^−/−^ or *C3aR* ^−/−^ mice were pretreated with 1 mg (40 mg/kg) of the IFNAR receptor blocking antibody MAR1-5A3 for one day prior to infection with WNV. At 72 hours, serum was collected and analyzed for proinflammatory cytokines (IL-1ß, IL-6, and TNF-α) and levels of glucose, AST, and ALT. The results are the average of at least two independent experiments for each genotype with n = 6 to 8 mice per group. Error bars and asterisks indicate SD and differences that are statistically significant (*, *P*<0.05; ***, *P*, <0.001), respectively.
(PDF)

Table S1 **Cytokine levels in serum of** ***Ifnar*** **^−/−^, CD11c Cre^+^** ***Ifnar*** **^f/f^, and Cre^−^** ***Ifnar*** **^f/f^ mice during CHIKV infection.** *Ifnar* ^−/−^, CD11c Cre^+^ *Ifnar* ^f/f^, and Cre^−^ *Ifnar* ^f/f^ mice (*n* = 13 for each group) were infected with 10 PFU of CHIKV. Seventy-two hours later, serum was collected and the concentration of cytokines was determined by bioplex assay. Mean (pg/ml) ± SD are shown and *P* values are compared to Cre^−^ *Ifnar* ^f/f^ mice. Data are pooled from two independent experiments. ns indicates data that are not statistically different.
(DOCX)
