# Supplementary material for: Deficient IFN Signaling by Myeloid Cells Leads to MAVS-Dependent Virus-Induced Sepsis
Source: PLoS Pathog. 2014 Apr 17;10(4):e1004086. doi: 10.1371/journal.ppat.1004086 (PMC3990718; doi:10.1371/journal.ppat.1004086)
Supplement: Figure S1 — Serum cytokine levels in CHIKV-infected mice. Ifnar −/−, CD11c Cre+ Ifnar f/f, and Cre− Ifnar f/f mice (n = 6 for each group) were infected with 10 PFU of CHIKV. Seventy-two hours later, serum was collected and the concentration of IL-1ß, IL-6, and TNF-α present was determined. Mean values and SD are shown. Asterisks indicate differences that are statistically significant (*, P<0.05). (PDF) [file ppat.1004086.s001.pdf]

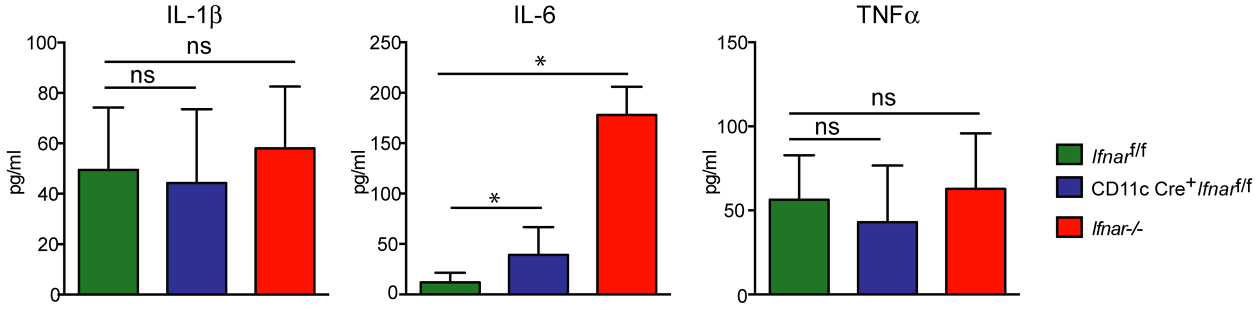

Figure S1. Serum cytokine levels in CHIKV-infected mice. *Ifnar*$^{-/-}$, CD11c Cre^{+} *Ifnar*$^{f/f}$, and Cre^{-} *Ifnar*$^{f/f}$ mice (n = 6 for each group) were infected with 10 PFU of CHIKV. Seventy-two hours later, serum was collected and the concentration of IL-1β, IL-6, and TNF-α present was determined. Mean values and SD are shown. Asterisks indicate differences that are statistically significant (*, $P < 0.05$).
