# Supplementary material for: Deficient IFN Signaling by Myeloid Cells Leads to MAVS-Dependent Virus-Induced Sepsis
Source: PLoS Pathog. 2014 Apr 17;10(4):e1004086. doi: 10.1371/journal.ppat.1004086 (PMC3990718; doi:10.1371/journal.ppat.1004086)
Supplement: Figure S2 — Levels of cytokines, ISGs, and chemokines in WT and KO DCs after WNV infection. qRT-PCR of WT, Mavs −/−, Ifnar −/−, and Mavs −/−×Ifnar −/− DKO DCs 24 and 48 hours after WNV infection. Relative RNA levels of IL-1ß, IL6, CCL5, IFIT1, IFIT2, and IFIT3 are shown and compared to uninfected cells. Mean values ± SD are shown. Asterisks indicate differences that are statistically significant (*, P<0.05; **, P<0.01). (PDF) [file ppat.1004086.s002.pdf]

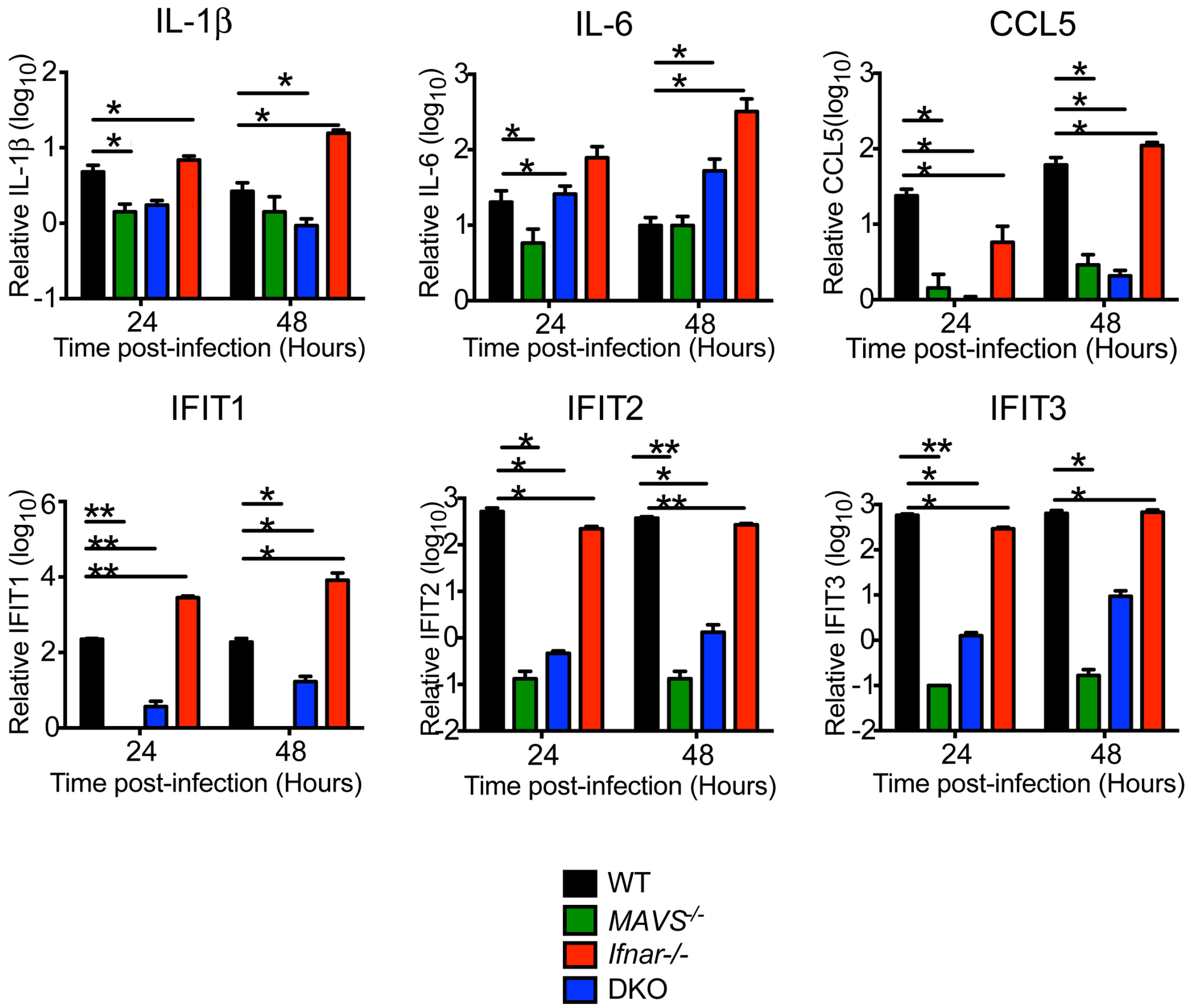

Figure S2. Levels of cytokines, ISGs, and chemokines in WT and KO DCs after WNV infection. qRT-PCR of WT, *Mavs*$^{-/-}$, *Ifnar*$^{-/-}$, and *Mavs*$^{-/-}$ x *Ifnar*$^{-/-}$ DKO DCs 24 and 48 hours after WNV infection. Relative RNA levels of IL-1β, IL-6, CCL5, IFIT1, IFIT2, and IFIT3 are shown and compared to uninfected cells. Mean values $\pm$ SD are shown. Asterisks indicate differences that are statistically significant (*, $P < 0.05$; **, $P < 0.01$).
