# Supplementary material for: Deficient IFN Signaling by Myeloid Cells Leads to MAVS-Dependent Virus-Induced Sepsis
Source: PLoS Pathog. 2014 Apr 17;10(4):e1004086. doi: 10.1371/journal.ppat.1004086 (PMC3990718; doi:10.1371/journal.ppat.1004086)
Supplement: Figure S3 — Effects of the NLRP3 inflammasome on cytokine induction. BMDCs (n = 3) from C57BL/6 or Nlrp3 −/− mice were pretreated with 25 µg/ml of the IFNAR receptor blocking antibody MAR1-5A3 or an isotype control GIR-208 for 30 minutes prior to infection with WNV and at 48 hours cells were collected. A. Western blot showing the expression of STAT1, the ISG IFIT2, the WNV protein NS3, and IL-1ß cleavage. B. Viral titers from the treated BMDCs were determined by a focus-forming assay. Data are shown as FFU per ml. Error bars indicate SD. C. Relative cytokine mRNA levels at 48 hours from WT or Nlrp3 −/− BMDCs infected with WNV after treatment with MAR1-5A3 or an isotype control MAb. Gene expression was measured by qRT-PCR and normalized to Gapdh levels. Data is displayed as the fold increase compared to untreated cells on a log2 scale. Data represent the average of three independent experiments. Error bars indicate SD. The limit of detection was assigned as a value log2 ΔΔCt of −2. D. The concentration of IL-1β, IL-6 and TNF-α in serum from the treated WT and Nlrp3− /− mice was determined by cytokine bioplex assay. Mean values ± SD are shown. E–G. WT, Nlrp3− /−, caspase-1/11 −/− or IL-1R −/− mice were pretreated one day prior to WNV infection with 1 mg (40 mg/kg) of the IFNAR receptor blocking antibody MAR1-5A3 or isotype control MAb (GIR-208) prior. At 72 hours after infection, serum was collected and analyzed for ALT (E), AST (F), and glucose (G). (PDF) [file ppat.1004086.s003.pdf]

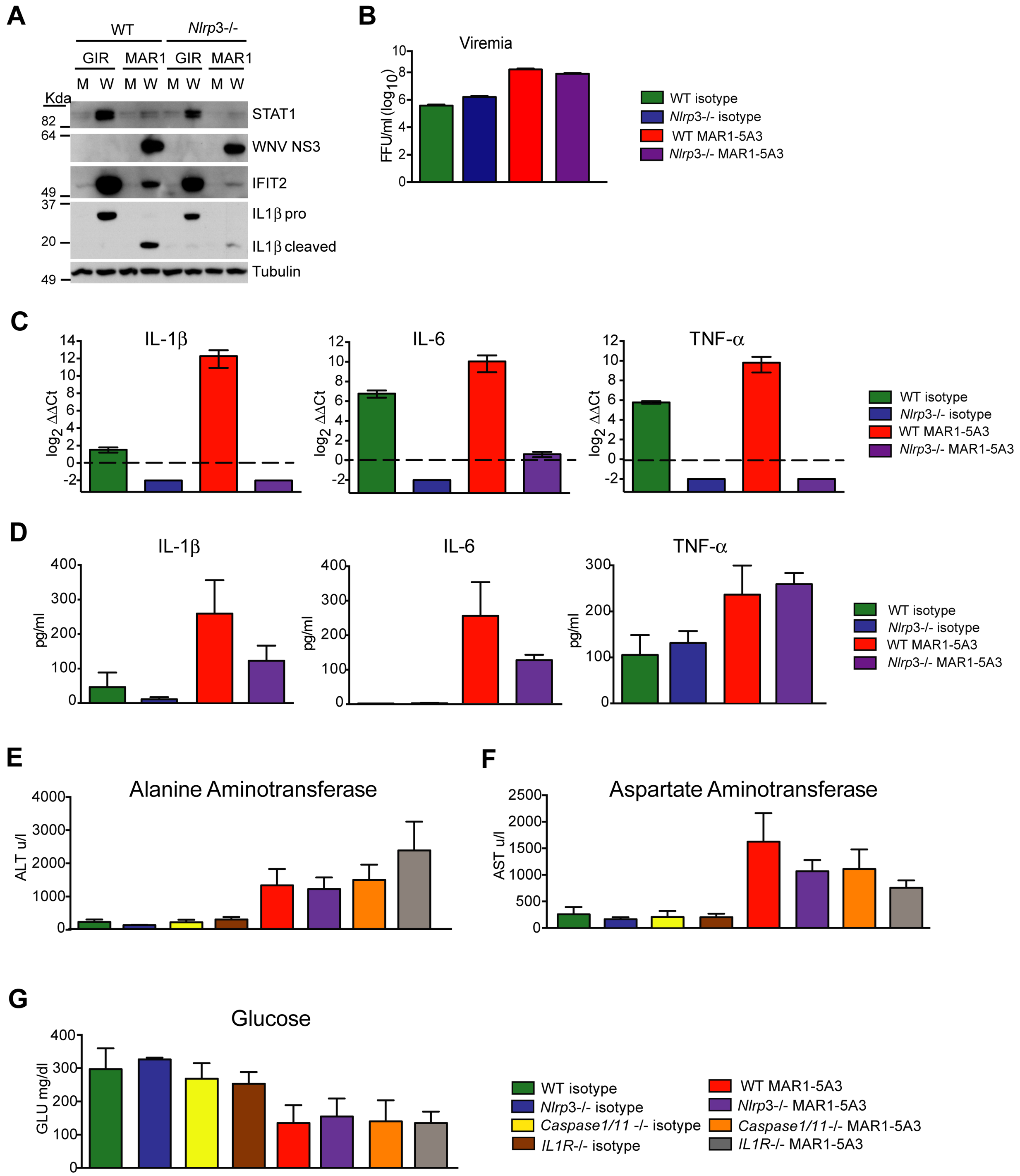

Figure S3. Effects of the NLRP3 inflammasome on cytokine induction. BMDCs (n = 3) from C57BL/6 or *Nlrp3*$^{-/-}$ mice were pretreated with 25 μg/ml of the IFNAR receptor blocking antibody MAR1-5A3 or an isotype control GIR-208 for 30 minutes prior to infection with WNV and at 48 hours cells were collected. **A**. Western blot showing the expression of STAT1, the ISG IFIT2, the WNV protein NS3, and IL-1β cleavage. **B**. Viral titers from the treated BMDCs were determined by a focus-forming assay. Data are shown as FFU per ml. Error bars indicate SD. **C**. Relative cytokine mRNA levels at 48 hours from WT or *Nlrp3*$^{-/-}$ BMDCs infected with WNV after

treatment with MAR1-5A3 or an isotype control MAb. Gene expression was measured by qRT-PCR and normalized to *Gapdh* levels. Data is displayed as the fold increase compared to untreated cells on a log2 scale. Data represent the average of three independent experiments. Error bars indicate SD. The limit of detection was assigned as a value log2 $\Delta\Delta$Ct of -2. **D**. The concentration of IL-1β, IL-6 and TNF-α in serum from the treated WT and *Nlrp3*$^{-/-}$ mice was determined by cytokine bioplex assay. Mean values $\pm$ SD are shown. **E-G**. WT, *Nlrp3*$^{-/-}$, *caspase-1/11*$^{-/-}$ or *IL-1R*$^{-/-}$ mice were pretreated one day prior to WNV infection with 1 mg (40 mg/kg) of the IFNAR receptor blocking antibody MAR1-5A3 or isotype control MAb (GIR-208) prior. At 72 hours after infection, serum was collected and analyzed for ALT (**E**), AST (**F**), and glucose (**G**).
