# Supplementary material for: Deficient IFN Signaling by Myeloid Cells Leads to MAVS-Dependent Virus-Induced Sepsis
Source: PLoS Pathog. 2014 Apr 17;10(4):e1004086. doi: 10.1371/journal.ppat.1004086 (PMC3990718; doi:10.1371/journal.ppat.1004086)
Supplement: Figure S4 — Impact of targeted deletion of mannose binding lectins, C4, or C3a receptor on cytokine levels, liver injury, and glycemia in mice lacking IFNAR signaling and infected with WNV. WT, Mbl-a −/−×Mbl-c −/− (MBL/AC−/−), C4 −/− or C3aR −/− mice were pretreated with 1 mg (40 mg/kg) of the IFNAR receptor blocking antibody MAR1-5A3 for one day prior to infection with WNV. At 72 hours, serum was collected and analyzed for proinflammatory cytokines (IL-1ß, IL-6, and TNF-α) and levels of glucose, AST, and ALT. The results are the average of at least two independent experiments for each genotype with n = 6 to 8 mice per group. Error bars and asterisks indicate SD and differences that are statistically significant (*, P<0.05; ***, P, <0.001), respectively. (PDF) [file ppat.1004086.s004.pdf]

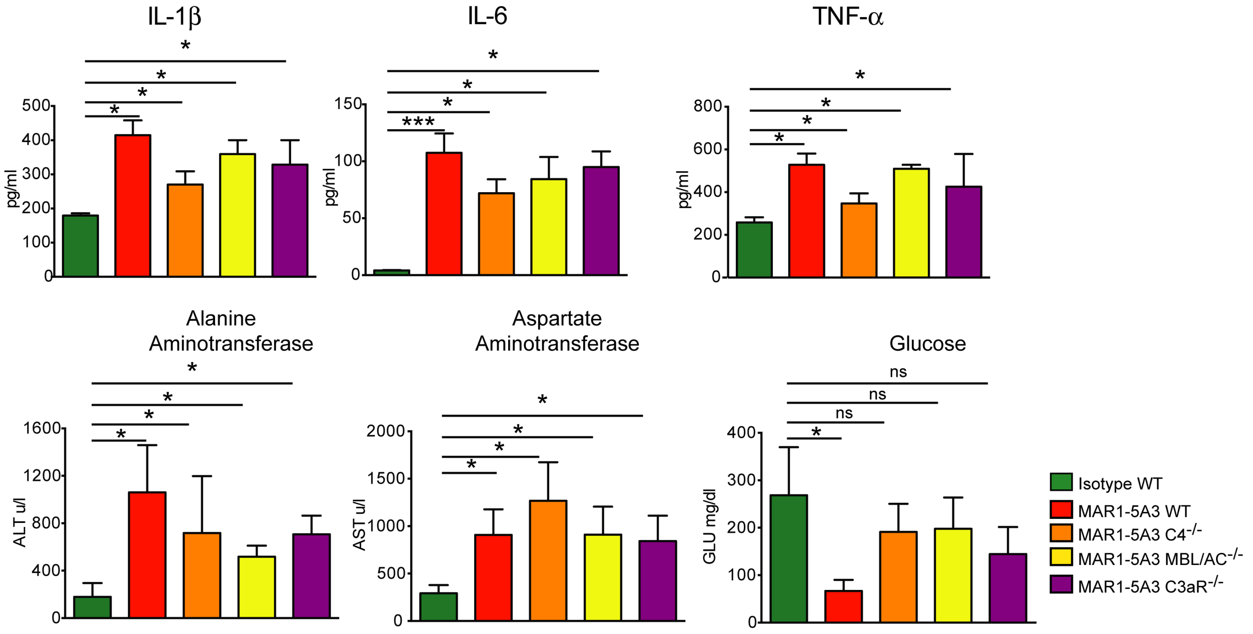

Figure S4. **Impact of targeted deletion of mannose binding lectins, C4, or C3a receptor on cytokine levels, liver injury, and glycemia in mice lacking IFNAR signaling and infected with WNV**. WT, *Mbl-a*$^{-/-}$ x *Mbl-c*$^{-/-}$ (MBL/AC$^{-/-}$), *C4*$^{-/-}$ or *C3aR*$^{-/-}$ mice were pretreated with 1 mg (40 mg/kg) of the IFNAR receptor blocking antibody MAR1-5A3 for one day prior to infection with WNV. At 72 hours, serum was collected and analyzed for proinflammatory cytokines (IL-1β, IL-6, and TNF-α) and levels of glucose, AST, and ALT. The results are the average of at least two independent experiments for each genotype with n = 6 to 8 mice per group. Error bars and asterisks indicate SD and differences that are statistically significant (*, P < 0.05; ***, P, < 0.001), respectively.
