# Supplementary material for: Deficient IFN Signaling by Myeloid Cells Leads to MAVS-Dependent Virus-Induced Sepsis
Source: PLoS Pathog. 2014 Apr 17;10(4):e1004086. doi: 10.1371/journal.ppat.1004086 (PMC3990718; doi:10.1371/journal.ppat.1004086)
Supplement: Table S1 — Cytokine levels in serum of Ifnar −/−, CD11c Cre+ Ifnar f/f, and Cre− Ifnar f/f mice during CHIKV infection. Ifnar −/−, CD11c Cre+ Ifnar f/f, and Cre− Ifnar f/f mice (n = 13 for each group) were infected with 10 PFU of CHIKV. Seventy-two hours later, serum was collected and the concentration of cytokines was determined by bioplex assay. Mean (pg/ml) ± SD are shown and P values are compared to Cre− Ifnar f/f mice. Data are pooled from two independent experiments. ns indicates data that are not statistically different. (DOCX) [file ppat.1004086.s005.docx]

**Table S1**. **Cytokine levels in serum of *Ifnar*^-/-^, CD11c Cre^+^ *Ifnar*^f/f^, and Cre^-^ *Ifnar*^f/f^ mice during CHIKV infection**

|  | Cre^-^*Ifnar*^f/f^ | | CD11c Cre^+^*Ifnar*^f/f^ | | | *Ifnar*^-/-^ | | |
| --- | --- | --- | --- | --- | --- | --- | --- | --- |
| Cytokine | pg/ml | SD | pg/ml | SD | *P* value | pg/ml | SD | *P* value |
| IL-1α | 8.33 | 4.08 | 9.46 | 3.74 | **ns** | 63.8 | 7.69 | 0.0357 |
| IL-1β | 49.5 | 102 | 44.3 | 29.2 | **ns** | 58.0 | 247 | **ns** |
| IL-2 | 6.25 | 0.35 | 7.43 | 0.40 | **ns** | 9.27 | 0.25 | **ns** |
| IL-3 | 4.10 | 0.71 | 4.67 | 1.40 | **ns** | 5.83 | 1.25 | **ns** |
| IL-4 | 2.15 | 0.35 | 2.50 | 0.36 | **ns** | 3.33 | 1.40 | **ns** |
| IL-5 | 3.40 | 0.00 | 3.77 | 1.16 | **ns** | 32.2 | 8.01 | **ns** |
| IL-6 | 11.9 | 9.65 | 39.3 | 27.4 | 0.02 | 178 | 27.9 | 0.0357 |
| IL-9 | 4.35 | 1.06 | 5.03 | 1.25 | **ns** | 11.0 | 1.10 | **ns** |
| IL-10 | 24.7 | 7.76 | 28.6 | 20.1 | **ns** | 34.2 | 3.55 | **ns** |
| IL-12p40 | 268 | 135 | 431 | 227 | **ns** | 1152 | 121 | 0.0357 |
| IL-12p70 | 12.7 | 1.77 | 19.3 | 3.19 | **ns** | 22.0 | 1.85 | **ns** |
| IL-13 | 5.05 | 2.08 | 8.40 | 0.35 | **ns** | 13.7 | 0.90 | **ns** |
| IL-17 | 41.9 | 18.1 | 63.5 | 17.4 | ns | 24.7 | 2.21 | ns |
| Eotaxin | 11.1 | 2.62 | 11.1 | 2.27 | **ns** | 24.4 | 2.85 | **ns** |
| g-CSF | 118 | 54.3 | 214 | 74.9 | **ns** | 4161 | 847 | 0.0357 |
| GM-CSF | 26.9 | 1.77 | 19.9 | 12.8 | **ns** | 26.0 | 6.93 | **ns** |
| IFN-γ | 18.0 | 8.28 | 15.0 | 12.2 | **ns** | 60.7 | 5.46 | 0.0357 |
| KC | 350 | 233 | 832 | 997 | **ns** | 4971 | 612 | **ns** |
| MCP-1 | 286 | 344 | 331 | 263 | **ns** | 671 | 109 | **ns** |
| MIP-1α | 62.0 | 19.2 | 57.8 | 25.5 | **ns** | 147 | 10.7 | 0.0357 |
| MIP-1β | 5.75 | 0.92 | 13.1 | 6.77 | **ns** | 16.8 | 3.57 | **ns** |
| RANTES | 270 | 167 | 279 | 147 | **ns** | 456 | 123 | **ns** |
| TNF-α | 42.9 | 33.7 | 56.3 | 26.5 | **ns** | 62.8 | 33.0 | **ns** |
